# ANIMA: Association network integration for multiscale analysis

**DOI:** 10.12688/wellcomeopenres.14073.3

**Published:** 2018-11-14

**Authors:** Armin Deffur, Robert J. Wilkinson, Bongani M. Mayosi, Nicola M. Mulder

**Affiliations:** 1 Department of Medicine, University of Cape Town, Cape Town, 7925, South Africa; 2 Wellcome Centre for Infectious Diseases Research in Africa, University of Cape Town, Cape Town, 7925, South Africa; 3 Francis Crick Institute, London, NW1 1AT, UK; 4 Imperial College London, London, W2 1PG, UK; 5 Computational Biology Division, Department Integrative Biomedical Sciences, IDM, University of Cape Town, Cape Town, 7925, South Africa

**Keywords:** Transcriptomics, complex networks, graph databases, data integration

## Abstract

Contextual functional interpretation of -omics data derived from clinical samples is a classical and difficult problem in computational systems biology. The measurement of thousands of data points on single samples has become routine but relating ‘big data’ datasets to the complexities of human pathobiology is an area of ongoing research. Complicating this is the fact that many publicly available datasets use bulk transcriptomics data from complex tissues like blood. The most prevalent analytic approaches derive molecular ‘signatures’ of disease states or apply modular analysis frameworks to the data. Here we describe ANIMA (association network integration for multiscale analysis), a network-based data integration method using clinical phenotype and microarray data as inputs. ANIMA is implemented in R and Neo4j and runs in Docker containers. In short, the build algorithm iterates over one or more transcriptomics datasets to generate a large, multipartite association network by executing multiple independent analytic steps (differential expression, deconvolution, modular analysis based on co-expression, pathway analysis) and integrating the results. Once the network is built, it can be queried directly using Cypher (a graph query language), or by custom functions that communicate with the graph database via language-specific APIs. We developed a web application using Shiny, which provides fully interactive, multiscale views of the data. Using our approach, we show that we can reconstruct multiple features of disease states at various scales of organization, from transcript abundance patterns of individual genes through co-expression patterns of groups of genes to patterns of cellular behaviour in whole blood samples, both in single experiments as well in meta-analyses of multiple datasets.

## Introduction

A frequent issue with bioinformatic analysis is the following scenario: a given dataset is analysed using various approaches in a linear workflow, in an attempt to extract biological features of interest from the data, often at different scales. For instance, a list of differentially abundant transcripts provides information on the system that differs from a list of co-expressed genes. While both such approaches are valid and provide independently useful information, these linear workflows do not not expose potentially informative relationships between different outputs; instead, multiple individual output items (plots, tables, etc.) are written to the output directory. However, multiple kinds of relationships, or associations, exist between entities of the same or different class in biological systems. For instance, two clinical variables might be correlated; the relationship is then expressed as correlation, its extent as the Pearson correlation coefficient, and the statistical significance of the relationship by a P-value, which may be corrected for multiple testing.

Such relationships can be discovered systematically in the analytic workflow. Typically, the result of such an analysis is written to disk in tabular form. However, a table of correlation statistics contains many non-significant entries, and therefore has a low signal to noise ratio, especially in large datasets. A more efficient approach would be to discard all results that fail to meet a pre-specified statistical cutoff and retain only the significant entries as a monopartite or bipartite network. Indeed, bipartite graphs are the dominant paradigm used to encode and represent information about *relationships* between entities of different classes. Bipartite graphs in systems biology and medicine have recently been reviewed ^1^, illustrating numerous use cases and applications as well as describing their mathematical properties. Once the decision has been made to store analytic results and their relationships as bipartite graphs (or other types of network), the next question is where and how to store the data. Cytoscape ^2^ is extensively used for visualising network data, mainly in biology. Large networks can be searched and subsetted, and basic network analysis can be performed. However, using Cytoscape for a network data store rather than a visualisation tool becomes cumbersome. Recently, graph databases have emerged as a type of noSQL database. Data in a graph database is stored as nodes and edges (relationships between nodes). Both nodes and edges can be assigned multiple properties with values. One such database is Neo4j, which is freely available. Neo4j databases are queried using Cypher, an intuitive query language, and provide computational access to scripting languages via dedicated APIs. In our view, graph databases are the ideal data store for network-type data.

Reproducibility of research is critical for scientific progress. Bioinformatic analyses can be very complex, but usually the results obtained depend strongly on the methods used, software versions, and even operating system. Typical narrative descriptions of analytic methods provide insufficient information to guarantee reproducibility. Workflow tools like Taverna ^3^ exist but are more suited to performing tasks that link together functionality offered by services (such as webservices). We prefer scripted workflows, where running and re-running the same script on the same data is guaranteed to produce identical results. However, providing the code used in analysis and the raw data does not guarantee reproducibility, as the computational environment in which the analysis is run can also influence the outcome of computations. Typically, this becomes an issue when the functionality of a software package changes between versions. Therefore, in addition to data and source code, one has to provide the exact configuration and package versions of all software involved in the project. In bioinformatics, this can become daunting very quickly, and leads to the well-known problem of “dependency hell” where not only the software packages need the right version, but also their dependencies. A solution for this is to package the entire computational environment in one or more “software containers”. The containerization platform Docker ^4^ is frequently used to fulfil this function and has enjoyed widespread adoption in reproducible research. This is exemplified by Nextflow ^5^, a workflow management system using Docker.

A recent paper presents GeNNet ^6^, which describes the rationale for scripted workflows and the use of graph databases in reproducible research. In this paper a scripted workflow in R ^7^, use of Neo4j to store data and the use of Docker for reproducibility is described. Other recent efforts in the very wide field of -OMICS data integration, research reproducibility and data integration include the Omics Integrator package ^8^ which takes a variety of -OMIC data (such as transcriptomic and proteomic) as input and identifies possible underlying molecular pathways using network optimization algorithms, NDEx ^9^, an online commons for sharing and searching biological networks.

Here we present ANIMA, Association Network Integration for Multiscale Analysis, a framework for producing and interrogating a multiscale association network, which allows summary and visualization of different, but simultaneously valid views of the state of the immune system under different conditions and at multiple scales. While ANIMA employs key strategies presented in the GeNNet paper, mainly “dockerisation”, scripted workflows in R and storage of relationships in a graph database, it differs in detail in implementation as well as complexity. In particular, we have defined novel functionality that allows the extraction of new information form the network structure in ANIMA, given an increased number of node types and increased complexity of the data model of the Neo4j database. In addition, GeNNet is implemented in a single container containing R and Neo4j, whereas ANIMA uses separate containers for each tool. 

ANIMA is targeted both at bioinformaticians and computational biologists who wish to reproduce the results presented or analyse their own data, as well as biologists who would interact with the system through the provided graphical user interface.

In its current form, ANIMA is best suited for investigating human immunity. The human immune system can be regarded as a complex adaptive system ^10^, even though it is integrated with the more complex system of the whole organism. A true systems view ^11^ of the immune system needs to account for the various aspects that characterize complex adaptive systems generally. This includes emergence, non-linearity, self-organization, noise, scaling, heterogeneity, a network architecture and preservation of context for individual observations ^12^. Whole blood is a “window” into the immune system ^13^, allowing a reasonably detailed assessment of the overall state of the immune system based on analysis of mRNA transcript abundance patterns from whole blood samples. Here we use the responses to three common infections (acute HIV infection, malaria and respiratory viral infections), as measured by transcript abundance patterns in whole blood, to demonstrate ANIMA functionality

## Methods

### Method overview

**Figure 1.**
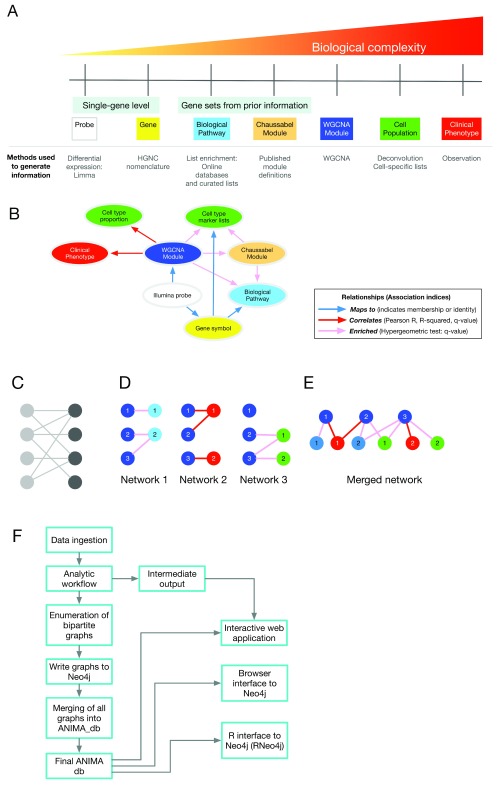
Method overview. ( **A**) Analytical approaches and biological complexity. This conceptualises the need for understanding biological systems at multiple scales. ( **B**) Relationships between output types ( **C**) A bipartite graph, with two classes of nodes connected by edges. ( **D**) The separate bipartite graphs, with one node type in common. ( **E**) Multipartite graph obtained after merging the three graphs in ( **D**). ( **F**) Outline of different steps in setting up and accessing the ANIMA database. Abbreviations: HGNC, HUGO Gene Nomenclature Committee; WGCNA, weighted gene co-expression network analysis.

ANIMA generates a multiscale association network (stored as a graph database) from multiple data types (expression data, clinical data and annotation data, e.g. biological pathways databases) by executing a comprehensive analytic workflow, enumerating bipartite graphs from the results, and merging all graphs into a single network. Figure 1 provides a conceptual overview of the multiscale analytics pipeline. As biological systems can be understood at multiple scales, our approach aims to integrate information across multiple scales which range from single genes to clinical phenotypes ( Figure 1A). This integration is summarised in the core data model for ANIMA ( Figure 1B) which represents relationships between various outputs of the analytic pipeline. Multiple bipartite graphs are generated from the outputs, and finally merged into a single data structure ( Figure 1 C–E), which is then accessed to gain novel information about the system. The various steps as implemented in the ANIMA_build script are illustrated in Figure 1F.

### Data ingestion and preparation

The first step in constructing an ANIMA database consists of accessing raw data, which consists of non-normalised Illumina BeadArray expression microarray data and clinical/phenotype data. A script (ANIMA_data.R) imports the data to the R workspace as well as the clinical data and creates a LumiBatch object of the imported data. Based on the experimental design, the data may be subsetted at this point. One or more LumiBatch objects are then saved to the project output folder for later re-use.

### Scripted workflow execution

The second step in constructing an ANIMA database consists of iterating over all datasets included in the analysis (each saved as an RData file), sequentially performing thirteen analytic tasks (A1–A13 in Table 1 and Figure S7; see Supplementary Data for details). Array normalization, probe filtering and differential expression analysis are frequently performed on transcriptomics datasets, but the other analyses tend to be performed in isolation. To our knowledge, this is the first transcriptomics workflow to combine WGCNA and Chaussabel modular analyses and integrate these with cellular-level approaches. The chosen approaches were selected based on their perceived popularity in the systems immunology literature, and selected annotation sources are biased towards immunology and cell-type specific data sets. This does not exclude the possibility of other approaches or annotation sets to be substituted in their place if the focus of investigation concerns other tissues or species of interest.

**Table 1.** List of analytic tasks used in constructing the ANIMA database. Tasks are referenced to Supplemental Figure S7, and described in Supplementary Methods under “Nodes in ANIMA”.

Task	Approach	Method	R package/implementation	Cutoff P value (BH corrected)	Reference
A1	Array data import and normalisation	variance stabilising transformation, quantile normalisation	lumi		15
A2	Probe filter	quality filter to remove non- informative probes from analysis	ReMoat, ReAnnotator		16, 17
A3	Differential expression	linear model/moderated t-test	limma	<0.05	18
A4	Estimate of cell- type proportions	Deconvolution by least- squares fitting	CellMix		19
A5	Chaussabel module expression	Published module definitions and custom R code			20, 21
A6	Chaussabel module differential expression	linear model/moderated t-test	limma	<0.05	18
A7	WGCNA module detection	Clustering of topological overlap matrix and dynamic tree cutting	WGCNA		14
A8	Chaussabel and WGCNA module annotation	List enrichment testing by hypergeometric test and multiple testing correction	WGCNA (UserListEnrichment); ReactomePA	<0.05	14, 22
A9	WGCNA module metrics	Differential expression, module AUC, signature enrichment	Custom code		
A10	Module eigengene correlations	Pearson or Spearman rank correlation	WGCNA, custom code	<0.05	14
A11	Phenotype data analysis	Univariate analysis (ordinal and numeric data)	Base R	<0.05	7
A12	Construction of bipartite graphs	Bipartite graphs from adjacency lists (based on list enrichment) or incidence matrices (based on correlation)	igraph		23
A13	Merging of all bipartite graphs	Graph union	Neo4j: cypher command *merge*		24

### Enumeration of bipartite graphs

Following the transcriptional analytic tasks, the algorithm constructs twenty-nine bipartite networks from a multi-scale analytic pipeline ( Figure 1A, C), combining the output, and the relationships between different classes of output approaches ( Table 1). Each network contains the associations between two distinct data types ( Figure 1B, D, Table 2, Table 3). The final association network is a result of graph union, merging all networks ( Table 3) on shared node types while retaining all edges ( Figure 1E). This results in a data structure that exposes the relationships between modular gene expression and higher-level phenomena, while retaining key probe- and gene-level information. Three types of association (with their respective association indices) are utilised, resulting in three distinct edge types in the final data structure (See Figure 1B, 1E, Table 2 and supplementary methods; Supplementary File 1). Weighted gene co-expression network analysis ^14^ (WGCNA) is the core analytic method in ANIMA as this is used to discover biological processes in the system of interest. Supplementary Figure S7 indicates the points where the bipartite graphs are enumerated.

**Table 2.** Types of associations used in the ANIMA database.

Association type	Association index	Intermediate result	Multiple testing correction
Correlation	Pearson R or Spearman rho	Incidence matrix	Yes
List enrichment	hypergeometric index	Adjacency list	Yes
Mapping	simple mapping	Adjacency list	Not applicable

**Table 3.** Bipartite graphs. Data types 1 and 2 (nodes in Neo4j) are defined in Supplementary Table S5, and indices refer to numbered bipartite graphs in Supplemental Figure S7 as well as their narrative description in Supplementary Data p.11–14. Data type names are given as implemented in the Neo4j database.

Index	Data type 1	Data type 2	Statistical method/ association type	Multiple testing correction	Cutoff (corrected P-value)	Implementation	Description and Biological implication	Reference(s)
1	PROBETYPE	SYMBOL	mapping	N/A	N/A	getSYMBOL function in lumi package	Reference mapping of all probes on the chip to genes (platform-specific)	14
2	PROBE	SYMBOL	mapping	N/A	N/A	topTable function in limma	Mapping of experiment-specific probes to genes	18
3	PROBE	wgcna	mapping	N/A	N/A	WGCNA	Indicates membership of specific probes in enumerated WGCNA modules	14
4 ^X^	PROBE	PROBE	Connectivity (weight in topological overlap matrix)	N/A	Maximum of 2000 per module	Custom function: at least the top 10 % of edges in the module network, capped at 2000	Subset of probes in WGCNA modules with high connectivity	14
5	SYMBOL	reactomePW	mapping	N/A	N/A	lumi, ReactomePA (uses reactome. db)	Mapping of gene symbols to reactome pathways in the current dataset	14, 22
6	SYMBOL	PalWangPW	mapping	N/A	N/A	WGCNA (uses PWLists included with WGCNA)	Mapping of gene symbols to a manually curated list of pathways in the current dataset	14
7	SYMBOL	ImmunePW	mapping	N/A	N/A	WGCNA (uses ImmunePathwayLists included with WGCNA)	Mapping of gene symbols to a manually curated list of immune pathways in the current dataset	14
8	SYMBOL	cellEx	mapping	N/A	N/A	WGCNA (uses BloodLists included with WGCNA)	Mapping of gene symbols to a manually curated list of cell-type specific genes in the current dataset	14
9	SYMBOL	cellEx	mapping	N/A	N/A	CellMix (HaemAtlas dataset)	Mapping of gene symbols to the Watkins, *et al.* list of cell-type specific genes in the current dataset	19, Supp. Ref 30
10	SYMBOL	cellEx	mapping	N/A	N/A	CellMix (Abbas dataset)	Mapping of gene symbols to the Abbas, *et al.* list of cell-type specific genes in the current dataset	19, Supp. Ref 11
11	wgcna	baylor (Chaussabel modules)	Hypergeometric test	BH	<0.05	WGCNA (UserListEnrichment function with Chaussabel module genes as input)	Enrichment of WGCNA modules for Chaussabel modules, allowing functional comparison of the two approaches	14
12	baylor (Chaussabel modules)	reactomePW	Hypergeometric test	BH	<0.05 *	WGCNA (UserListEnrichment function with Reactome genes as input)	Enrichment of Chaussabel modules for Reactome pathways, allowing functional annotation of the module	14, 22
13	baylor (Chaussabel modules)	PalWangPW	Hypergeometric test	BH	<0.05 **	WGCNA (UserListEnrichment function with a custom list of pathway genes as input)	Enrichment of Chaussabel modules for a manually curated list of pathways, allowing functional annotation of the module	14
14	baylor (Chaussabel modules)	ImmunePW	Hypergeometric test	BH	<0.05 ***	WGCNA (UserListEnrichment function with a custom list of immune pathway genes as input)	Enrichment of Chaussabel modules for a manually curated list of immune pathways, allowing functional annotation of the module	14
15	wgcna	reactomePW	Hypergeometric test	BH	<0.05 *	WGCNA (UserListEnrichment function with Reactome genes as input)	Enrichment of WGCNA modules for Reactome pathways, allowing functional annotation of the module	14, 22
16	wgcna	PalWangPW	Hypergeometric test	BH	<0.05 **	WGCNA (UserListEnrichment function with a custom list of pathway genes as input)	Enrichment of WGCNA modules for a manually curated list of pathways, allowing functional annotation of the module	14
17	wgcna	ImmunePW	Hypergeometric test	BH	<0.05 ***	WGCNA (UserListEnrichment function with a custom list of immune pathway genes as input)	Enrichment of WGCNA modules for a manually curated list of immune pathways, allowing functional annotation of the module	14
18	baylor (Chaussabel modules)	cellEx	Hypergeometric test	BH	<0.05	WGCNA (UserListEnrichment function, uses **BloodLists** included with WGCNA)	Enrichment of Chaussabel modules for a manually curated list of cell-type specific genes, allowing interpretation of cellular context of the module	14
19	baylor (Chaussabel modules)	cellEx	Hypergeometric test	BH	<0.05	WGCNA (UserListEnrichment function, uses **HaemAtlas** list included with Cellmix)	Enrichment of Chaussabel modules for a manually curated list of cell-type specific genes, allowing interpretation of cellular context of the module	14, 19, Supp. Ref 30
20	baylor (Chaussabel modules)	cellEx	Hypergeometric test	BH	<0.05	WGCNA (UserListEnrichment function, uses **Abbas** list included with Cellmix)	Enrichment of Chaussabel modules for a manually curated list of cell-type specific genes, allowing interpretation of cellular context of the module	14, 19, Supp. Ref 11
21	wgcna	cellEx	Hypergeometric test	BH	<0.05	WGCNA (UserListEnrichment function, uses **BloodLists** included with WGCNA)	Enrichment of WGCNA modules for a manually curated list of cell-type specific genes, allowing interpretation of cellular context of the module	14
22	wgcna	cellEx	Hypergeometric test	BH	<0.05	WGCNA (UserListEnrichment function, uses **HaemAtlas** list included with Cellmix)	Enrichment of WGCNA modules for a manually curated list of cell-type specific genes, allowing interpretation of cellular context of the module	14, 19, Supp. Ref 30
23	wgcna	cellEx	Hypergeometric test	BH	<0.05	WGCNA (UserListEnrichment function, uses **Abbas** list included with Cellmix	Enrichment of WGCNA modules for a manually curated list of cell-type specific genes, allowing interpretation of cellular context of the module	14, 19, Supp. Ref 11
24	wgcna	cellprop	Pearson correlation	BH	<0.05 ***	WGCNA (WGCNA::cor function)	Correlation of WGCNA module eigengenes with estimated cell-type proportions (as output using functions in the CellMix package)	14
25	CELL	cellEx	mapping	N/A	N/A	Mapping of consensus cell type names to cell type names used in the **BloodLists** dataset included with WGCNA)	Convenience mapping that facilitates searching for cell types in the ANIMA database regardless of the name of the cell type in the original gene marker list	14
26	CELL	cellEx	mapping	N/A	N/A	Mapping of consensus cell type names to cell type names used in the **HaemAtlas** dataset included with CellMix)	Convenience mapping that facilitates searching for cell types in the ANIMA database regardless of the name of the cell type in the original gene marker list	19, Supp. Ref 30
27	CELL	cellEx	mapping	N/A	N/A	Mapping of consensus cell type names to cell type names used in the **Abbas** dataset included with CellMix)	Convenience mapping that facilitates searching for cell types in the ANIMA database regardless of the name of the cell type in the original gene marker list	19, Supp. Ref 11
28	CELL	cellprop	mapping	N/A	N/A	Mapping of consensus cell type names to cell type names used in the **Abbas** dataset included with CellMix)	Convenience mapping that facilitates searching for cell types in the ANIMA database regardless of the name of the cell type in the original gene marker list	19, Supp. Ref 11
29	wgcna	pheno	Pearson correlation	BH	<0.05 ***	WGCNA (WGCNA::cor function)	Correlation of WGCNA module eigengenes with a numeric matrix of clinical and other phenotype data	14

*a minimum of three and a maximum of ten results were included in the ANIMA database ** a minimum of four and a maximum of ten results were included in the ANIMA database *** results were reviewed algorithmically; in the case of no hits for enrichment at the specified cutoff, less stringent criteria were applied (See Supplementary information). In all cases, the corrected P-value was included as a property of the relationship, allowing filtering of the database entries. ^X^ the PROBE-PROBE network is a monopartite correlation network and not a bipartite graph Abbreviations: BH, Benjamini-Hochberg multiple testing correction

Network construction relies on three principles. Firstly, mathematical operations on data are performed, independent of prior knowledge (e.g. WGCNA networks). This aspect of the approach is completely unsupervised. The second process involves the testing of hypotheses, (e.g. determination of differential transcript abundance and differential co-expression of transcripts, requiring knowledge of phenotype/trait classes for the samples). Finally, the results of the first two processes are integrated in various ways with prior biological knowledge. The result is a collection of statistically robust analytic results and various associations between them and known biology, in the form of a multiple bipartite graphs.

### ANIMA database

The twenty nine graphs share node types between them, as indicated in Supplemental Figure S7b, which describes the “data model” for the graph database. As each bipartite graph is enumerated, it is added to the Neo4j database instantiated at the start of the build process. Nodes and relationships are added using the “MERGE” command, ensuring that nodes are not added in duplicate. At the end of the build script run, a large graph has been generated, stored as a Neo4j graph database. This is referred to as the *ANIMA* database, which makes the overall network structure as well as the individual nodes and edges accessible for further analysis (see supplementary methods; Supplementary File 1).

### Accessing ANIMA

After network construction, information in the graph is accessible and utilized to expose new information not present in any of the individual steps. The key to making the ANIMA database useful lies in the use of functions and web applications (see supplementary methods; Supplementary File 1) that query this large multipartite graph and return visualization of relationships or tables of nodes and/or links (associations between nodes). This has been implemented in several R functions which underpin a Shiny web application called *ANIMA REGO*.

### Constructing an ANIMA database from user data

This paper and associated files provided as supplementary data allow the reconstruction of the particular ANIMA database presented here. However, it warrants emphasis that we are describing a *method* here, and that the provided datasets can be exchanged for a user’s own data. The scripts at present only support Illumina microarray data, although future work aims to add support for other microarray platforms and RNAseq data.

In order to construct a new ANIMA database, the following is required in the *source_data* folder:

1. Non-normalised expression data (.txt format)
2. Clinical/phenotype data (csv format)
3. A file named “matrixPD” in csv format or similar that contains the names of the datasets to be analysed, as well as the classes of each of the variables included in the phenotype data, distinguishing numeric and categorical data types, and flagging certain variables as identifiers.
4. A file named “questions.R” which is an R script specifying a list which contains, for each dataset, multiple variable assignments required in the running of the analysis. Essentially, this file encapsulates the information required to execute the experimental design.
5. A file named “setlist.R”, another R script, providing high level project information, the url of the graph database, the various studies the datasets are drawn from, as well as the identifiers of data sets that contain matched samples, which enable additional analytic functionality.

With the required files in place, the scripts ANIMA_data.R and ANIMA_build.R are run in sequence, and the end state should be a new ANIMA database based on the user data. The supplementary information provides full replication instructions.

### Using ANIMA

The novelty and value in ANIMA lies in its three broad approaches to data interpretation: Firstly, it allows detailed, multiscale investigation of a single dataset with a focused research question where two phenotype classes are compared **(multiscale class comparison)**. Secondly, as datasets are stratified by default using two variables (typically one identifying two disease classes, and the other either a potential confounder (e.g. sex), or a second biologic variable of interest, (like co-infection with a second pathogen), we can examine the interaction of the second variable with the first using a factorial study design **(factorial analysis)**. Finally, multiple datasets, and multiple conditions can be meaningfully compared and contrasted to identify similarities and differences **(meta-analysis)**, both at cellular and modular level.

### Application areas

In its current state, the ANIMA approach is optimised to address questions in immunology, but other application areas that may be tissue type specific (neuroscience, oncology) are also possible, but will require addition of annotation libraries and lists of cell-type specific genes.

## Results

### Example study: data sets

We analysed three publicly available microarray datasets using the ANIMA pipeline and toolset (ArrayExpress/GEO identifiers: E-GEOD-29429, E-GEOD-34404/GSE34404, E-GEOD-68310/GSE68310). The first compares a cohort of subjects with acute HIV infection to healthy controls ^25^, the second compares symptomatic malaria to asymptomatic controls in children from a malaria-endemic region ^26^, and the last compares the host response in early symptomatic viral respiratory infections ^27^. Where the datasets contained samples from multiple timepoints, we restricted the analysis to healthy controls and the first disease timepoint. The respiratory virus infection dataset contained samples from subjects with infections other than influenza or rhinovirus; we excluded those from this analysis. Figure S1 ( Supplementary File 1) shows the experimental design for the factorial analysis in *limma* for each of the datasets, together with the numbers of samples in each of the individual groups. Each of the datasets also included clinical data, which was integrated in the analysis.

### The ANIMA network

The result of the script that builds the ANIMA network is a large, mostly connected, multipartite graph. Figure S2 ( Supplementary File 1) shows a subgraph of this network (for dataset **HIVsetB**, edge 5).

### Searching the ANIMA network using network paths and filtering on node and edge properties

In the most direct approach, the large ANIMA graph in the Neo4j database can be searched directly from a web browser using the Cypher Query Language (CQL).

Consider the following query:



                        MATCH (ph:pheno)-[r1]-(n:wgcna {square:'HIVsetB',edge:5})-[r2]-(p:PROBE)-[r3]-(s:SYMBOL) WHERE r1.weight > 0.6 AND p.logfc > 2 RETURN *
                    


The query language combines commands and functions (e.g. “MATCH”, “WHERE”) with a typographic representation of nodes and relationships, where a node is indicated with round brackets **( )** and a relationship is indicated by dashes either side of a set of square brackets **-[ ]-**. Taken together, the query above conducts a search (or graph traversal) for a specific graph pattern matching MATCH WGCNA modules for the HIV positive vs control comparison in the **HIVsetB** dataset (n:wgcna {square:'HIVsetB',edge:5}) whose module eigengene (ME) is strongly correlated with any clinical variable (Pearson R > .6) (ph:pheno)-[r1]-; WHERE r1.weight > 0.6, and also returning the genes mapping to probes within those modules -[r2]-(p:PROBE) that have high levels of over-abundance in HIV-1 infected individuals AND p.logfc > 2.

**Figure 2.**
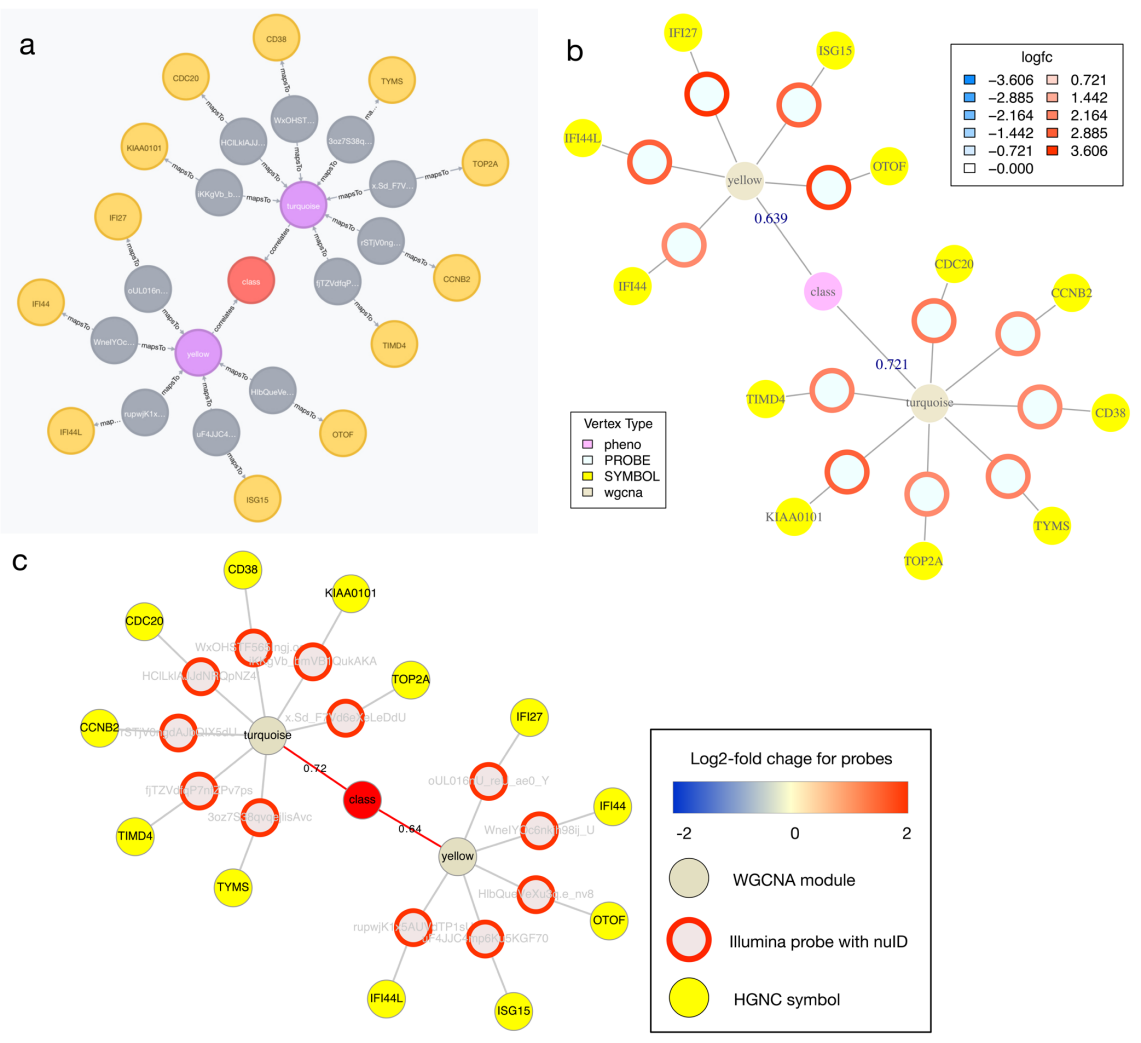
Visualising Cypher query results. Relationships between nodes extracted from the ANIMA database using a Cypher query applied to the **HIVsetB** data (N _HIV_=30, N _Controls_=17, see Figure S1; Supplementary File 1). Shown are two WGCNA modules that contain probes with increased transcript abundance in acute HIV infection and whose module eigengene is positively correlated with disease class (an ordinal variable). ( **A**) Result from native browser interface for Neo4j. ( **B**) Result plotted from within an R session connected to the ANIMA database, using the *igraph_plotter* function. Log _2_-fold change values for the individual probes are shown by coloured rings; values are shown in the legend. ( **C**) The same result, visualized in Cytoscape, taking advantage of the *igraph_plotter* function to export node and edge lists for easy import into Cytoscape. Links/edges are annotated with Pearson correlation coefficients where applicable.

In addition to the standard web browser interface for Neo4j, into which the above query can be directly entered and returned in the browser window ( Figure 2A), we also provide a function in R ( igraph_plotter) that returns the network found, and plots this within R ( Figure 2B), exports the network as node and edge lists for import in other software like Cytoscape ( Figure 2C), or returns the result as an *igraph* ^23^ object for further manipulation within R. (A file ANIMA_styles.xml is included in the common folder supplied with the source code; this is a Cytoscape stylesheet that reproduces the colouring shown in the Figure 2C)

More sophisticated methods of using ANIMA are described next. These utilise both bespoke R functions as well dynamic interactivity provided in the Shiny web interface (see below).

### Approach 1: Multiscale class comparison

**Figure 3.**
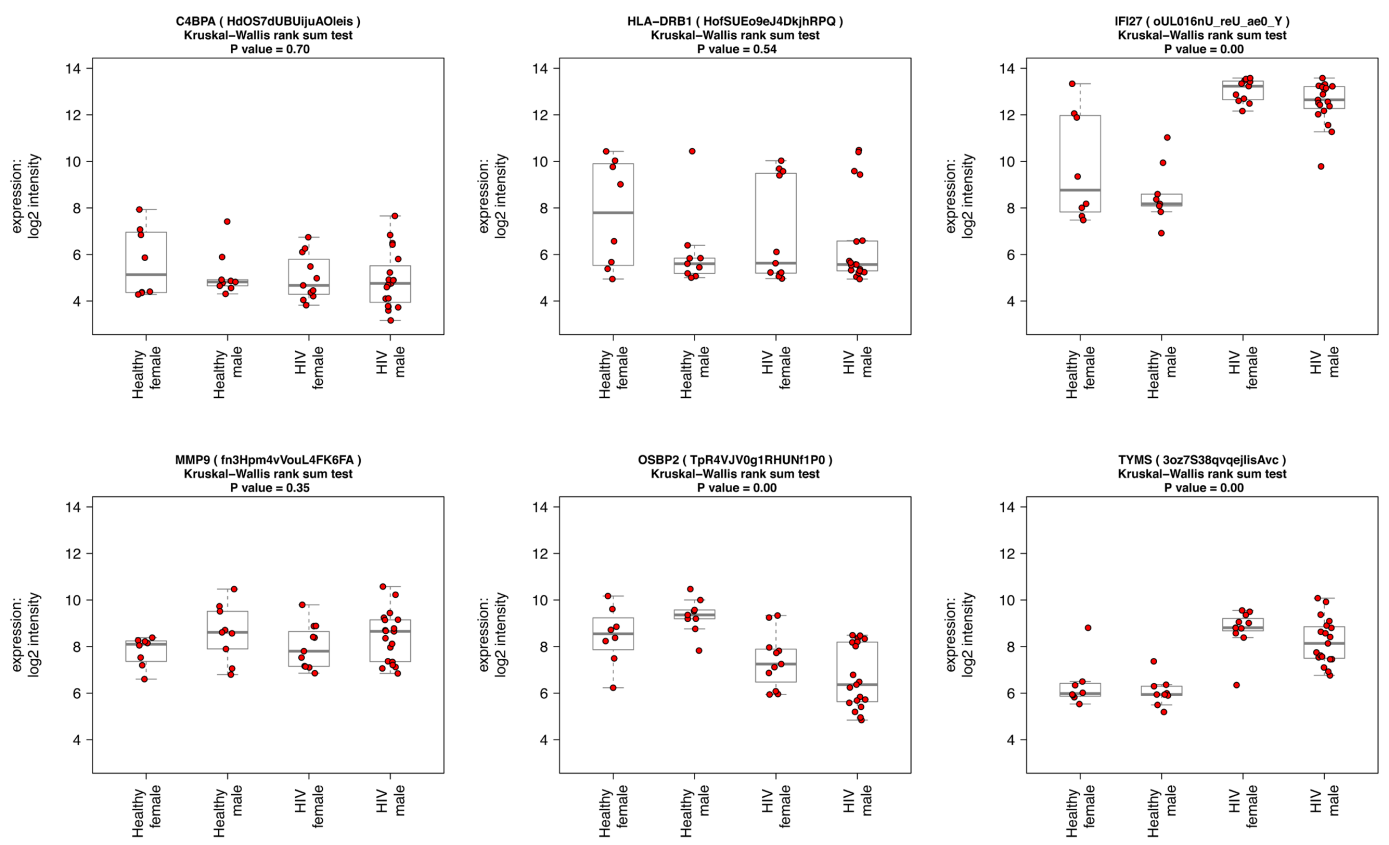
Visualising individual probe-level expression data. Box-and-whisker plots showing normalized, log _2_-transformed probe-level expression data for six selected genes, obtained by a custom function in R in four groups: Healthy female, N = 8, Healthy male, N = 9, acute HIV female, N = 11, acute HIV male, N = 19; data from **HIVsetB** dataset. Gene (and probe nuIDs for disambiguation) are given for reference; the y-axis shows log2 scale normalized intensity values. Box and whisker plots show median, interquartile range, and range. Outliers are defined as values that lie beyond the whiskers, which extend to maximally 1.5 X the length of the box. Individual datapoints are superimposed in red on the box-and-whisker plots. The four groups are compared using Kruskal Wallis rank sum test, and the P-value for the comparison is shown in the plot title. Results for individual pairwise comparisons are not shown.

***Accessing individual transcript abundance levels in multiple conditions***. It is useful to view transcript abundance patterns of specified probesets, for instance to compare microarray data to RT-PCR validation data, or to investigate the behaviour of groups of biologically related genes in various conditions. We provide an interface for this in ANIMA. The user can submit a search string (in the form of a regular expression) containing gene names (HUGO Gene Nomenclature Committee (HGNC) symbols), and box-and whisker plots for the results are returned. In the original paper on acute HIV infection the authors discuss a gene set of six conserved genes that appear at multiple timepoints in an inferred regulatory network of viral set point ^25^. We show the normalized expression data stratified by HIV status and sex in the two datasets included in the HIV analysis for these six genes ( Figure 3). In the paper on acute viral respiratory infection, IFI27 and PI3 are identified to differ between acute influenza A and human rhinovirus infections. In influenza, IFI27 is upregulated and PI3 downregulated relative to human rhinovirus. The malaria study replicated prior knowledge of differential transcript abundance for C1QB, MMP9, C3AR1, IL18R and HMOX1; we show similar results for these transcripts. Supplementary Table 1 lists the results of differential expression analysis for the above transcripts in the three conditions, providing validation of data at individual transcript level.

**Figure 4.**
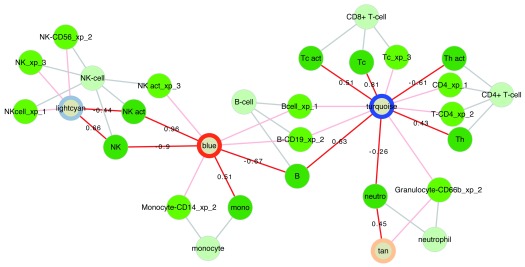
Cell associations of WGCNA modules. Relationships of WGCNA modules and different cell types in the **respInf** dataset (Day 0 acute influenza, N = 46 vs baseline healthy samples, N = 48, see Figure S1; Supplementary File 1). Shown are WGCNA modules whose expression correlates with specific cell-type proportions (dark green, edges annotated with Pearson correlation coefficient *R*) *and* that are enriched for the genes specific to that cell type (medium green, suffixes xp_1-3 indicate the respective gene list on which the cell assignments were based, see Supplementary methods). The classes of cells are indicated in light green. The modules are annotated with coloured rings representing the difference in median eigengene values between cases and controls (diffME, see Supplementary methods); blue indicates modules which are under-expressed, and red indicates modules that are over-expressed in cases relative to controls. WGCNA module names are (arbitrarily) based on colours as per the convention of the WGCNA package, and modules were not renamed manually.

***Functional annotation of WGCNA modules***. An important question is what do WGCNA modules represent, given that these are groups of genes that co-vary across samples, but that can differ dramatically in size. Instead of searching only for evidence of co-regulation of expression by transcription factors, one should consider other causes of this co-variance. We propose the notion of WGCNA modules representing biological processes that may be regulated at different scales. For instance, a group of transcripts will co-vary across samples if these transcripts are expressed (predominantly) in a single cell type, and the proportions of this cell type varies between samples ( Figure 4). Another group of transcripts may be expressed in multiple cell types, but represents a concerted transcriptional program executed in response to a specific stimulus, such as interferon-alpha stimulating the expression of a specific group of interferon-regulated genes (see below). Generally, we determine the function of WGCNA modules based on all statistically significant associations with pathways and cells.

**Figure 5.**
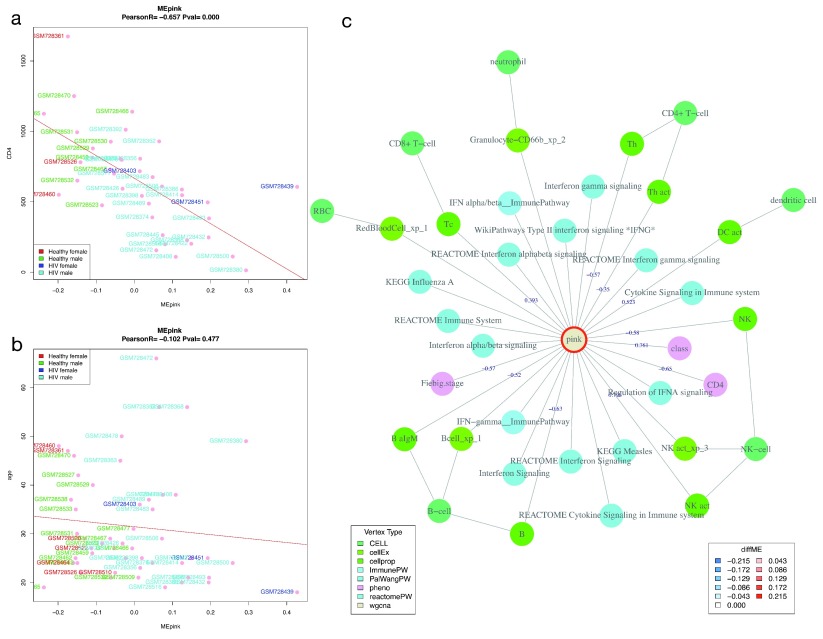
Correlation of module eigengenes with clinical variables. Shown is the Pearson correlation of the *pink* module eigengene with CD4 count (cells/microlitre) ( **A**) and with age (years) ( **B**) in acute HIV (N = 28) vs healthy controls (N = 23) in the **HIVsetA** dataset. Study subject IDs are used as point labels, and coloured as indicated in the legend. Plot titles show the Pearson coefficient R and the associated P-value. ( **C**) WGCNA module annotation obtained from the Neo4j database for the *pink* module. Edges are labelled with the correlation coefficient (R) where applicable. Note that the same coefficient is obtained for CD4 count as in panel **A**. Legends are shown for vertex type and diffME (a measure of differential co-expression (see Supplementary methods), i.e. the extent that the module eigengene median varies between two classes). Abbreviations: **diffME**, differential module eigengene.

***Relationship of modules to clinical variables***. We performed Pearson correlation of WGCNA module eigengenes (ME) and clinical variables (described in supplementary methods). Figure 5 shows correlation of the *pink* ME with age and CD4 count in the **HIVsetA** data set. Clearly, the *pink* module is significantly associated with CD4 count, an association that is independent of age. Further investigation shows that this module is linked to interferon signalling, and probably expressed in a variety of cells. This agrees with our understanding of acute HIV infection, which is associated with a robust type-I interferon response and an acute drop in CD4 count ^28^.

**Figure 6.**
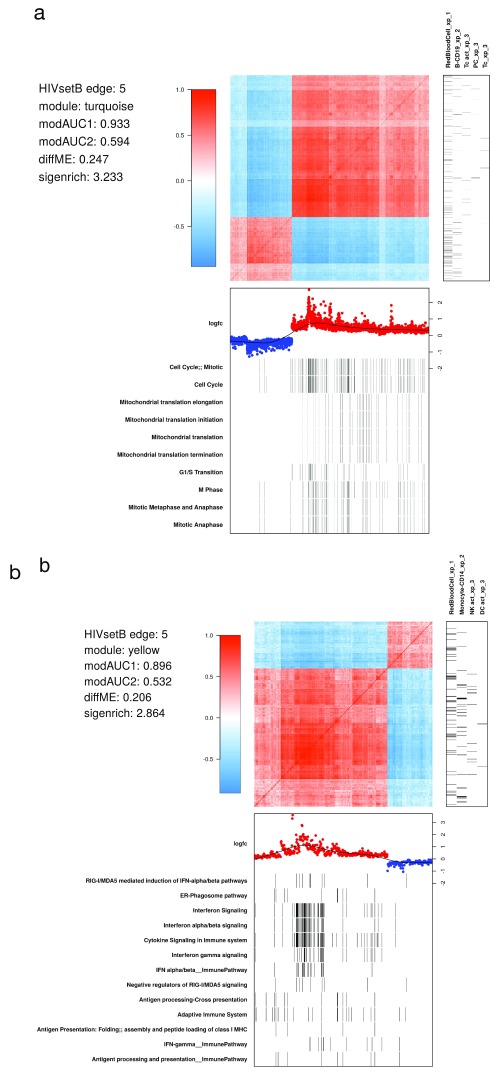
WGCNA module structure. ( **A**) Correlation matrix of all probes in the *turquoise* module in the HIVsetB dataset (N _HIV_=30, N _Controls_=17, see Figure S1; Supplementary File 1). Colours in the heatmap represent Pearson correlation coefficients, ranging from -1 to 1, as indicated by the legend. The module is enriched for lymphocyte-specific genes (right annotation panel) as well as cell cycle/mitosis associated genes, suggesting that various lymphocyte subsets in acute HIV infection are actively proliferating. (bottom annotation panel). Log _2_-fold change values refer to differential transcript abundance in acute HIV relative to healthy controls. ( **B**) Correlation matrix of all probes in the *yellow* module in the HIVsetB dataset. It is enriched for innate cell genes as well as interferon signaling, suggesting that innate immune cells are in an interferon-induced state. Additional annotation information is provided to the left of the heatmap. The parameters *modAUC1*, *modAUC2*, *diffME* and *sigenrich* are defined in Supplementary methods. The plot is generated using a custom R function ( *mwat*).

***Investigating the structure of WGCNA modules***. WGCNA modules are groups of co-expressed transcripts. Demonstrating the extent and direction of correlation of their constituent probes, and their relationships to biological pathways requires sophisticated visualization ( Figure 6). We showcase the example using the **HIVsetB** dataset and focus on two modules. The *turquoise* module shows the coordinated action of genes involved in cell division, relating to the cell proliferation in the lymphoid compartment. The *yellow* module is clearly related to interferon signalling; of interest here is that within this module there is a group of transcripts highly correlated with each other, all related to interferon signalling, suggesting that these transcripts may all be downstream of a single regulatory factor. Also clear from the figure is that this module is not limited to a single cell type, but rather to innate immune cells in general.

**Figure 7.**
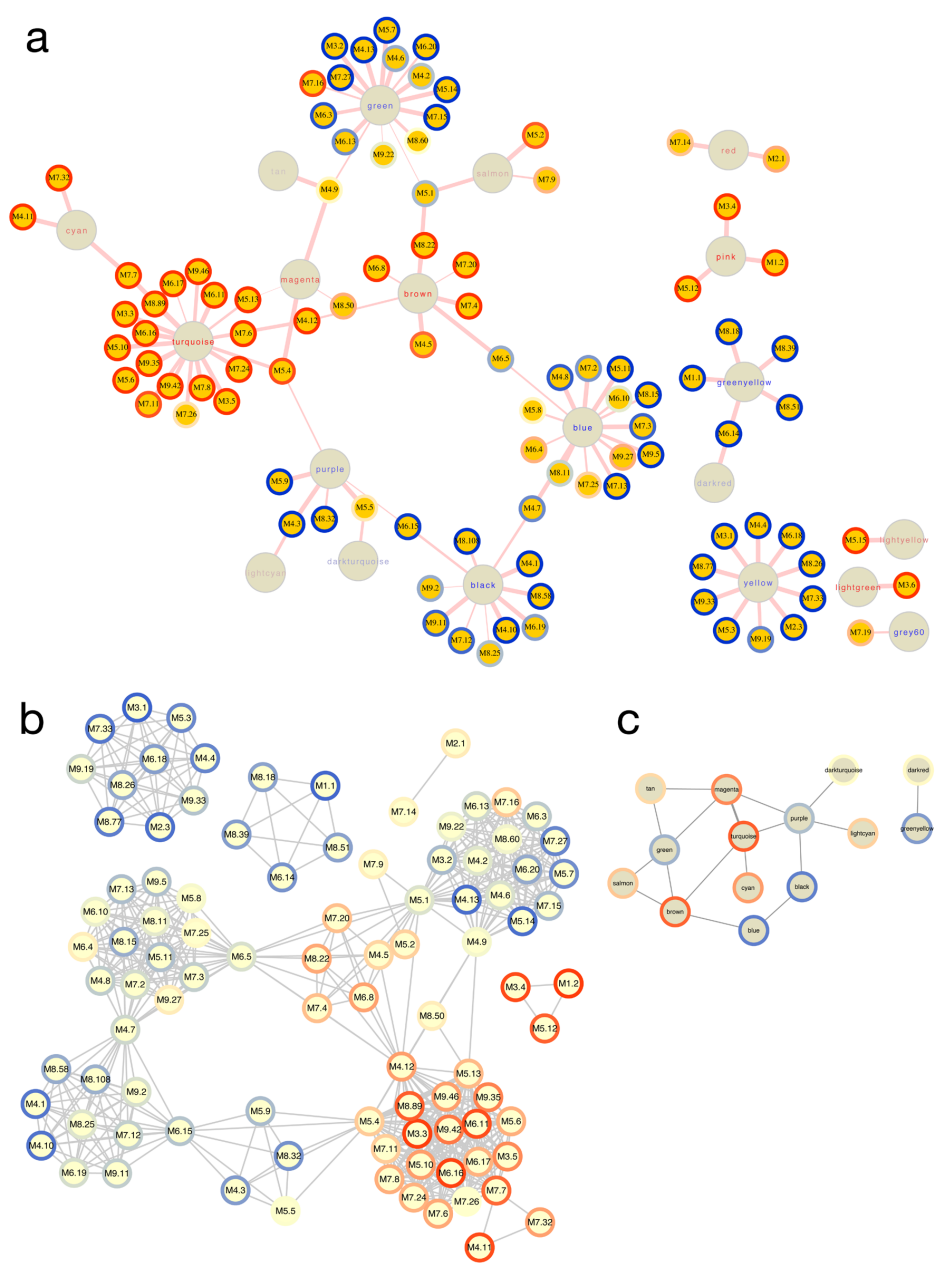
Relationships between WGCNA and Chaussabel modules. ( **A**) Bipartite graph of the two module types based on the hypergeometric association index in the **HIVsetA** dataset (acute HIV, N = 28 vs healthy controls, N = 23). Strikingly, Chaussabel modules tend to have the same direction of differential expression (indicated by the rim colour of the Chaussabel modules, red indicating up-regulation in acute HIV, and blue indicating downregulation) as WGCNA modules they map to, indicated by the label colour of the module. ( **B**) Projection 1 of ( **A**), showing relationships between Chaussabel modules based on shared WGCNA modules; dense cliques of modules are observed. ( **C**) Projection 2 of ( **A**), showing relationships between WGCNA modules based on shared Chaussabel modules. All associations (hypergeometric test) shown are corrected for multiple testing, BH-corrected P-value < 0.05. All outputs were generated using the *igraph_plotter* function, exporting vertex and edge tables of the bipartite graph and the two projections and importing these into Cytoscape.

***Relationships between module-based approaches***. Both WGCNA and the modular approach pioneered by Chaussabel, *et al.* ^17^ rely on clustering of similarity matrices to derive modules. A key difference in these methods in ANIMA is that the Chaussabel modules are pre-defined, whereas the WGCNA modules are derived from the transcriptional data under study. It is therefore interesting to discover the relationships between these two approaches. Figure 7A shows the bipartite network of WGCNA and Chaussabel modules derived from the **HIVsetA** dataset, as defined by the hypergeometric index. Figures 7B and 7C show the two projections of the bipartite graph. The hypergeometric index is not the only way associations between the two module types can be demonstrated; for instance, a more indirect association can be inferred when a WGCNA and Chaussabel module map to the same biological pathway. It is clear from the plots that only a subset of the list of Chaussabel modules associates with WGCNA modules in any given condition.

***Deconvolution***. An important question in analysing transcription data from complex tissues is whether differences in transcript abundance are attributable to transcriptional regulation in one or more cell types, or to changes in the composition of the overall leukocyte populations.

Figure S3 (Supplementary File 1) shows the results for the **HIVsetB** dataset, and Table S2 ( Supplementary File 1) shows the results of non-parametric statistical testing for differences in median cell-type proportions, per cell-type and class comparison with uncorrected P values as well as P-values corrected for multiple testing using the Benjamini-Hochberg procedure; these were encoded as parameters *diffP* and *diffQ* of the *cellprop* node type in the ANIMA database. Neutrophils were the most abundant cell-type estimated from the array data. The proportions of activated NK cells and CD8+ T cells were significantly elevated in acute HIV infection, and proportions of B cells and CD4+ T cells were reduced, in line with published observations ^29^.

**Figure 8.**
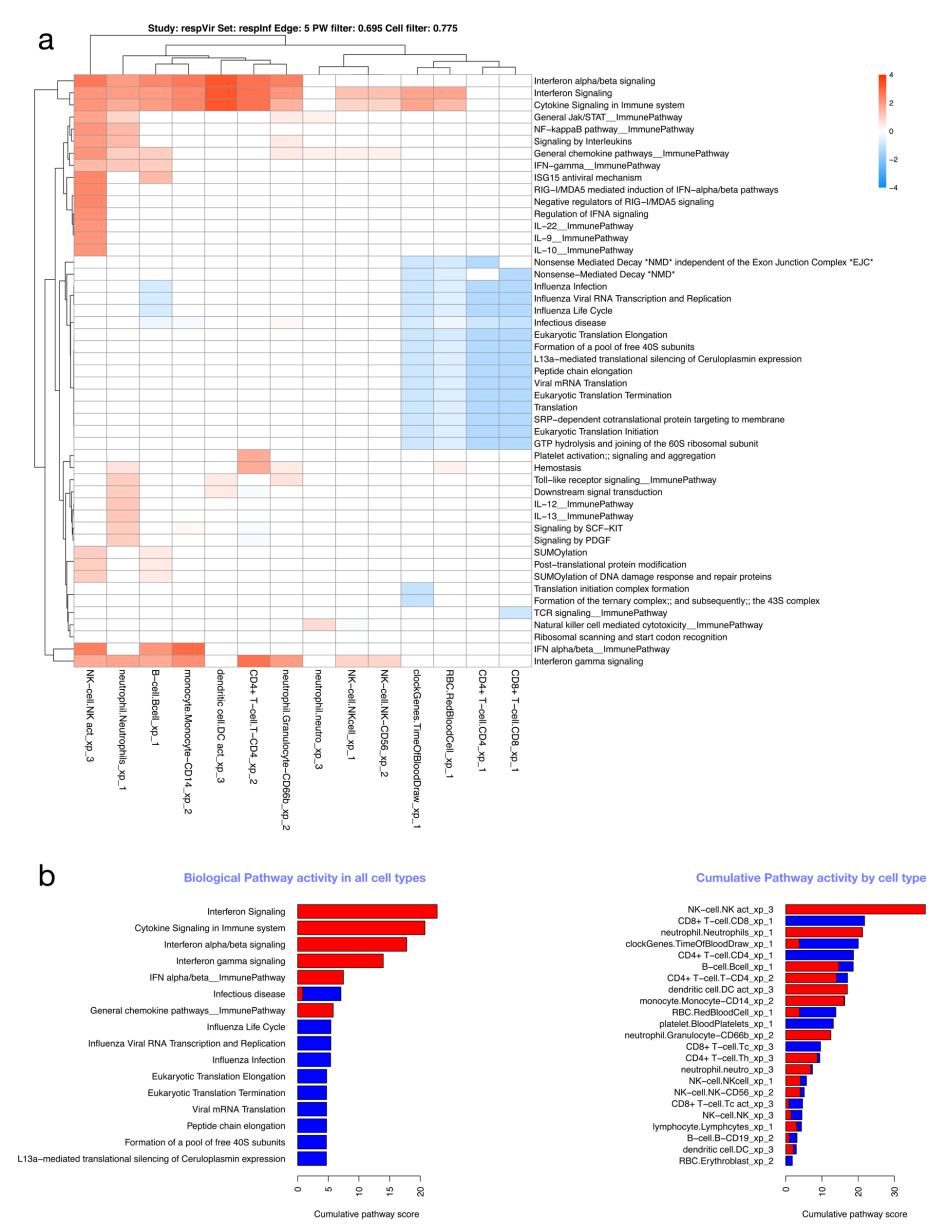
Cell/pathway activity matrix. ( **A**) Cell/pathway activity matrix for all cell-types for the **respInf** dataset (Day 0 acute influenza, N = 46 vs baseline healthy samples, N = 48, see Figure S1). The clustered heatmap shows pathway activity scores representing the mean log-2 fold change for all probes in the pathway for a particular cell type (see Supplementary methods). There is a clear interferon response in multiple cell types, as well as down-regulation of other pathways associated with translation. ( **B**) Barplots highlighting the most highly differentially regulated pathways (left panel, determined by row sums of matrix in **A**), and cells with highest levels of differential expression (right panel, determined by column sums of matrix in **A**). In all cases, up- and downregulated pathway scores are kept separate.

***Virtual cells: an estimate of functional phenotypes of different immune cell types***. Given that the immune response is mediated by different types of cell, we attempted to re-create “virtual cells” based on the assumption the genes that co-vary in terms of transcript abundance across samples with that of cell-type specific genes are expressed in that particular cell type. With these relationships, we generated virtual cells (probe co-expression matrices annotated with biological pathways and probe differential expression data). Figure S4 (Supplementary File 1) shows two cells (B-cells and neutrophils) in acute HIV infection; both characterized by interferon signalling. Given the “virtual cells” and their functions we can compare pathway-level transcript abundance in different cell types by creating a matrix of cell types and pathways, with each entry representing the “pathway activity” for a given cell and pathway combination. To illustrate this, we show replication of the finding of NK-cell activation in acute influenza infection ( **respInf** dataset, Figure 8 A,B), and in addition provide more detail on which pathways are probably up- or downregulated in these and multiple other cells.

### Approach 2: Factorial analysis

**Figure 9.**
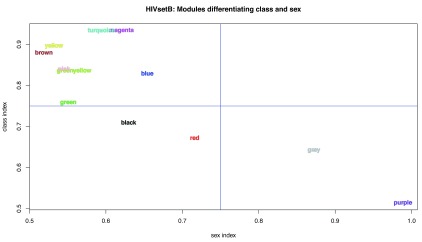
WGCNA module indices. Plot of module indices representing the area-under-the-ROC curve for the two classes for all WGCNA modules in the **HIVsetB** dataset (N _HIV_=30, N _Controls_=17, see Figure S1; Supplementary File 1). The indices are named per the variable they aim to differentiate (disease class or sex). The class index corresponds to the modAUC1 variable and the sex index corresponds to modAUC2. These indices are calculated form the module eigengenes and given class assignments using functions from the *rocr* package. See text and Supplementary methods for details.

***Modules driven by sample class or sex***. Our main interest in investigating transcriptomic datasets is to identify molecular and cellular processes that drive, or at least are associated with, specific phenotypic traits of the samples. Therefore, the experimental design for the differential abundance analysis and the probe filtering steps prior to WGCNA module discovery were both designed to highlight processes associated with two factors: disease class and sex; the former because this is the basis of the research question for the three studies, and the latter because sex has a distinct influence on immune system function ^30^. We developed a novel approach to quantify the association of the WGCNA module expression with these two factors (See supplementary methods; Supplementary File 1). Figure 9 shows that, in the case of acute HIV infection, most modules are associated with the disease process ( **HIVsetB** dataset), but that one module ( *purple*) is strongly associated with sex. Figure S5 (Supplementary File 1) shows the module eigengenes for the *yellow* and *purple* modules, demonstrating differential class associations for WGCNA modules; this finding would have been missed had the data not been stratified by both HIV infection status and sex. Table S4 shows the module statistics for this dataset.

Additional modules were identified that associated with neither sample class nor sex. These represent biologic processes that manifest in heterogeneity of the sampled population. Figure S6 (Supplementary File 1) plots the study subjects in the HIV infection data set ( **HIVsetA**) on two axes represented by two module eigengenes.

### Approach 3: Meta-analysis of multiple datasets

**Figure 10.**
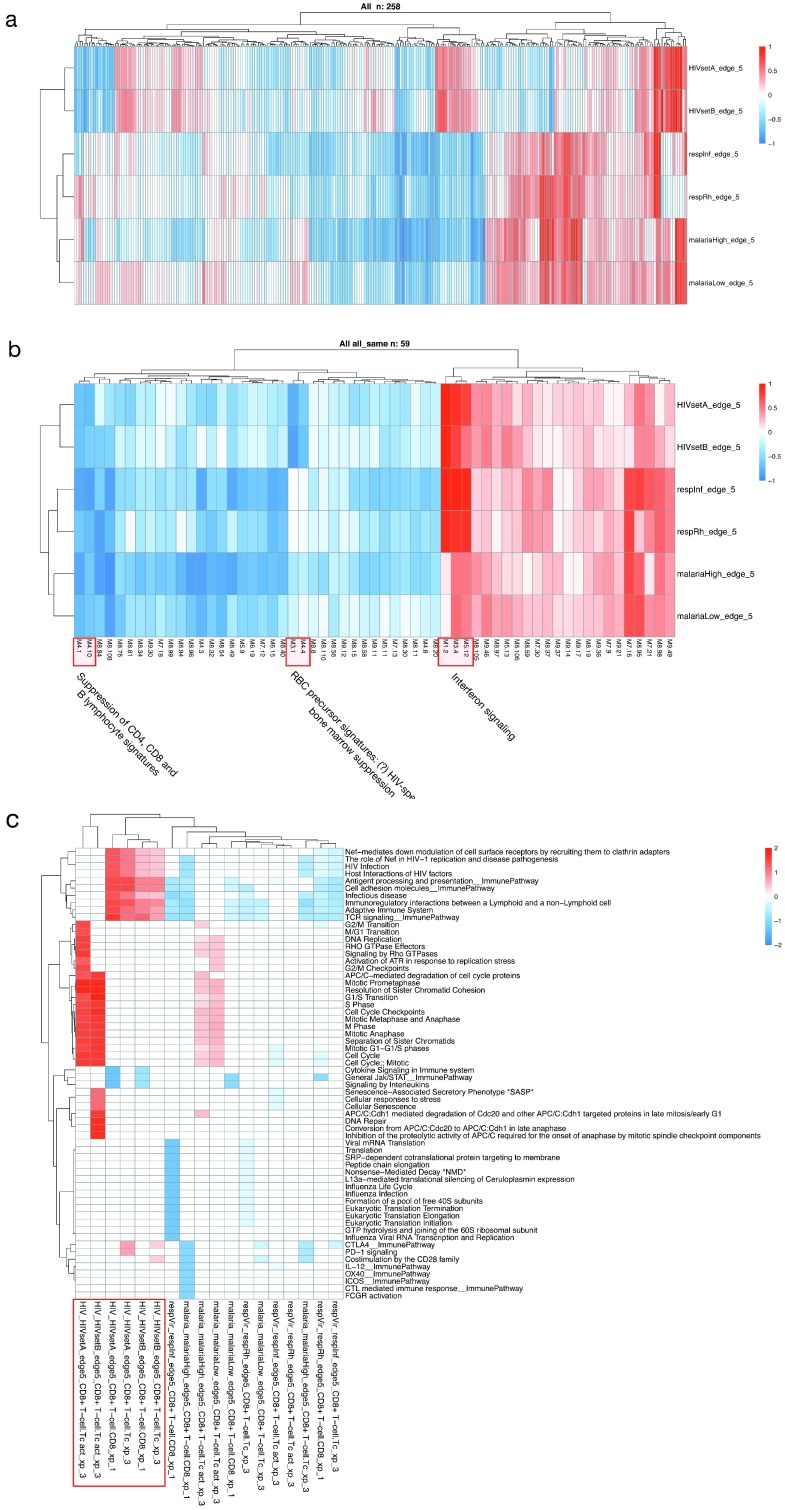
Meta-analysis of transcriptional and cellular patterns. ( **A**) All 258 Chaussabel modules plotted as a heatmap in all six datasets. ( **B**) The subset of modules all expressed in the same direction. Three module groups of interest are identified. ( **C**) Cell/pathway activity matrix for a single cell type (CD8 + T cell) based on three celltype-gene lists (xp1, xp2, xp3, see Supplementary methods) in all three conditions. Activity in CD8+ T cells in HIV all cluster together, and differ from both malaria and respiratory infections. Cell labels are constructed by [condition]_[dataset]_[comparison]_[cell class]_[cell type]_[gene list].

***Module-level meta-analysis***. Meta-analysis of multiple related expression datasets can lead to insights not available from analysis of any single datasets, and can highlight common patterns of transcript abundance across different conditions, or meaningful differences across highly common conditions. We implemented the approach pioneered ^20^ and refined ^21^ by Chaussabel, *et al.* to perform modular transcriptional repertoire analysis ^31^ on the six datasets. This approach is particularly suited to meta-analysis, as the composition of the modules is always identical. Figure 10 shows modular patterns for the six datasets in a clustered heatmap as well as the subset of modules with similar expression patterns across the six data sets, demonstrating universal patterns in the immune response to infection. Supplementary Table S3 ( Supplementary File 2) lists module functions based on all significant enrichment associations for the modules in Figure 10B. Universal upregulation of interferon-related modules particularly stand out, as does the suppression of modules associated with CD4+ T cells, CD8 T cells and B cells.

***Meta-analysis at the cell-type level***. A second approach to meta-analysis was implemented using virtual cells based on WGCNA modules. Here we compared the pathway scores in a single or several cell types across multiple conditions. For instance, comparing the CD8 T-cell response in acute HIV, acute viral respiratory infection, and symptomatic malaria, we find that proliferation of activated CD8+ T cells characterizes acute HIV infection, and to a lesser extent symptomatic malaria infection. In contrast, there is a suppression of CD8 T cell activity in blood in other conditions, due in part to a reduction in CD8+ T-cell proportions in whole blood ( Figure 10C).

### Runtime statistics and output

The build script produces a file called session_output.txt. This file captures various runtime statistics, as well as some textual output. This specific ANIMA database was created on a 2013 Mac Pro, 8 cores, 64 GB RAM, with 7 cores and 55 GB RAM allocated to Docker. The build phase took 34.3 hours to complete. Once the database is built, the various components of the Shiny web interface compute and render within seconds, depending on resources available.

## Discussion

Systems immunology aims to understand the complex web of relationships between immune system components (cells, cytokines, effector molecules and other mediators) in immune-mediated disease states. Much progress has been made in single-cell techniques that yield large amounts of information, e.g. single-cell RNAseq and mass cytometry. These approaches however are expensive. In contrast, RNAseq or microarray analysis of complex tissue samples (like blood) in principle contain information on the transcriptomic state of all cells present in the sample and is thus an unbiased approach. The main difficulty with this lies in the interpretation of the data, and in many cases a complexity-reducing analysis approach is employed, where the focus is placed on differentially expressed genes. Other approaches based on co-expression analysis often fail to explain the drivers of the co-expression patterns.

We demonstrate, using a novel method of aggregating information obtained from clinical and microarray data, an ability to reconstruct many aspects of the immune response, and to discuss this not in the language of probes, genes and signatures, but rather as coordinated biological processes and the cellular context for these processes, allowing the generation of hypotheses at multiple scales. Our multiscale class comparison approach can be used to validate findings from individual papers, for instance the intense NK-cell activation described in influenza ^27^. In contrast to other approaches, e.g. PARADIGM ^32^, which rank pathways in specified conditions, we utilise the multiscale association information to infer states of immune cells (virtual cells) and providing an interface to compare such cell states within and between datasets. Pathway and cell-activity ranking are provided as a summary of multiple cell states.

Using our factorial approach, we can begin to dissect inter individual heterogeneity in transcriptional patterns from transcriptional patterns that are in a causal relationship with defined factors. Application of the two meta-analysis approaches allows comparison of arbitrary datasets to detect similarities and differences at modular and cellular levels. An interesting and somewhat unexpected finding is that acute symptomatic malaria and acute respiratory viral illnesses are more similar to each other than to acute HIV infection, another viral illness. Despite these differences, we demonstrate that, at least for these three rather different infections, a broadly similar pattern of transcriptional module activity can be described.

In summary, ANIMA is both a robust implementation of various well-regarded analytic paradigms in microarray analysis, as well as a framework for integrating these various methods to expose relationships at multiple scales and render these computationally accessible.

## Future work

ANIMA is an open-ended project. Future work may include a demonstration that the associations in the final network are robust to choice of package. This requires a technical comparison study incorporating multiple packages with the same goals. A priority of ongoing development is improving the ease with which users can import their own data, as well as adding support for more microarray platforms and RNAseq data. Customisation of ANIMA at this stage requires modification of the source code to explicitly add additional functionality. With modularization of code, we hope to make this process easier. The scope of ANIMA can be widened by including support for more tissue types and organisms. Integration of protein-protein interaction data using existing databases is planned. Finally, we envision a repository where completed ANIMA projects may be archived as well as hosted.

## Software availability

Source code (R code, dockerfiles) are available at GitHub at https://github.com/adeffur/anima under a CC BY-NC 4.0 license

An archive of source code releases linked to github is available on Zenodo ( http://doi.org/10.5281/zenodo.1163398 ^33^)

The docker image used to implement ANIMA is available at Docker hub ( https://hub.docker.com/r/animatest/anima/) with the tag v3.3.3

The same image has been archived and is available on Zenodo ( https://doi.org/10.5281/zenodo.1161475 ^34^)

## System requirements

1. Minimum system requirements for installing and running Docker must be met. Docker is freely available for macOS, Microsoft Windows and various Linux distributions. See https://docs.docker.com/install/ for further information
2. To run the full ANIMA pipeline, including the build phase, Docker must be provisioned with at least 50GB RAM, and 6–8 cores
3. To run the ANIMA web application, at least 8GB of system RAM is required, but 16GB is preferable.

## Data availability

Array Express: **Whole Blood Transcriptional Response to Early Acute HIV**, E-GEOD-29429

Gene Expression Omnibus: **The genomic architecture of host whole blood transcriptional response to malaria infection**, GSE34404

Gene Expression Omnibus: **Host transcriptional response to influenza and other acute respiratory viral infections – a prospective cohort study**, GSE68310

A single zip archive will all data, and clinical and other metadata has been archived on Zenodo; for reproducibility purposes it is advised that this archive is used as described in the manual ( Supplementary File 3) ( https://doi.org/10.5281/zenodo.1161380 ^35^).

## Supplementary material

1. **Supplementary File 1: ANIMA_method_SUPP_submission_WOR.7z**
This document contains supplementary methods, additional figures and tables, implementation and replication information.
2. **Supplementary File 2: Table S3 New.xlsx**
Supplementary Table S3 in Excel format.
3. **Supplementary File 3: ANIMA_manual_v1.0.0.pdf**
This document contains full instructions to replicate the ANIMA pipeline and web application, as well as screenshots describing the functionality of the web application.

## Pre-print

A preprint of this work has been submitted to biorxiv (doi: https://doi.org/10.1101/257642 ^36^)
