# Supplementary material for: ANIMA: Association network integration for multiscale analysis
Source: Wellcome Open Res. 2018 Nov 14;3:27. Originally published 2018 Mar 12. [Version 3] doi: 10.12688/wellcomeopenres.14073.3 (PMC6134339; doi:10.12688/wellcomeopenres.14073.3)
Supplement: Supplementary file 3 [file wellcomeopenres-3-16261-s0002.tgz › f215372b-606f-4f75-92d5-cffb167061c3.pdf]

Manual/ Tutorial (DRAFT)

ANIMA: Association Network Integration for Multiscale Analysis

Armin Deffur[1*], Robert J Wilkinson[1,2,3,4], Bongani M Mayosi[1] and Nicola Mulder[5]

[1]Department of Medicine, University of Cape Town, South Africa

[2]Wellcome Centre for Infectious Diseases Research in Africa, University of Cape Town, South Africa

[3]Francis Crick Institute, London, NW1 2AT, United Kingdom

[4]Imperial College London, W2 1PG, United Kingdom

[5]Computational Biology Division, Department Integrative Biomedical Sciences, IDM, University of Cape Town, South Africa

* Corresponding author at: Department of Medicine, Old Main Building, Faculty of Health Sciences, University of Cape Town, Anzio Road, Observatory, 7925, South Africa

E-mail address: A.Deffur@uct.ac.za

Running Title: ANIMA: Association Network Integration for Multiscale Analysis

Keywords: Microarray, Data integration, graph database, complex networks

Replication instructions

Execute the following steps to reproduce the analysis

1. Install Docker. The exact method and requirements may differ depending on the host operating system. See https://www.docker.com/community-edition
2. Get the *anima* Docker image
    a. Directly from Docker Hub via commandline: `docker pull animatest/anima:v3.3.3`
    b. Download the archived version from Zenodo (DOI: 10.5281/zenodo.1161476), unzip, and install with `docker load < anima_image_archive.tar`
3. Choose the ANIMA install location and install the source code. Installing in the root of the current user's home folder is *highly* recommended. Should another location be chosen, edit the docker-compose.yml file appropriately after downloading the source code. Initiate the install by getting the source code for ANIMA:
    a. Using git from the home folder: `cd ~; git clone https://github.com/adeffur/ANIMA`
    b. The ANIMA folder can also be created manually, and the contents of the github repository downloaded from Zenodo (DOI:)
4. Get the source data, metadata, and project specific scripts from Zenodo
    a. Download **ANIMA_method.zip** from Zenodo (DOI: 10.5281/zenodo.1161381) and extract in ANIMA folder
5. Optional step: Download the complete output of the ANIMA_build.R script from Zenodo. *This step can be omitted, but then the output folder and Neo4j database*

*have to be built by running the appropriate scripts; this requires at least **55GB** RAM to be available to Docker (see below).*

   a. Download **version_2017-07-20_18_47_11.zip** from Zenodo (DOI: 10.5281/zenodo.1156355), and extract to ~/ANIMA/output/build (create folders as required)

6. Find the IP address of the Docker daemon. This may differ on different systems. The easiest way to do this on the commandline, and only needs to be done once:
   a. Run the following command: `docker run --net=host codenvy/che-ip`
   b. The codenvy image will be installed and the container run; the IP address of the docker daemon is the only output. This IP address needs to be inserted on line 2 in the file ~/ANIMA/ANIMA_method/data/source_data/setlist.R. for instance for the IP address **192.168.65.3** the line should read: `graphstring<-"http://192.168.65.3:10474/db/data/"`
7. Start ANIMA using docker-compose script. Two variants of the docker-compose.yml file are provided in ~/ANIMA/ANIMA_method, each in a separate folder: *compose_big* and *compose_small*. The scripts differ based on how much memory is allocated to Docker. The compose_small script requires 16GB of system RAM but can be modified to run successfully on systems with as little as 8GB of RAM. The higher amount of RAM in the compose_big variant is required for running ANIMA_build.R. due to the nature of the script, and an issue with Linux memory management in the *anima* container.
   a. On the commandline, navigate to ~/ANIMA/ANIMA_method/compose_small
   b. Start ANIMA with: `docker-compose -p method up -d`

c. Assuming that the *anima* Docker image has already been installed, the script will pull additional images from Docker hub the first time it is run: neo4j:2.3, portainer/portainer:latest and google/cadvisor. The latter two images are utilities to monitor container use and activity, and are not required for ANIMA to function

d. Check that all containers are running using `docker ps`

e. After starting ANIMA, navigate to http://localhost:10787 in the browser. The login screen for RStudio should be present. Log in with username = rstudio and password = rstudio.

f. In a separate browser window navigate to http://localhost:10474/browser/ in the browser. The web interface for Neo4j should be available. The database is empty at this point.

8. With the complete computational environment, source data, metadata and scripts in place, the first step of replication requires the creation of RData files that store the expression data and metadata

    a. From the RStudio interface, open the file Home/scripts/ANIMA_data.R and run the script. After completion, the RData files for each of the data subsets are saved in ~/ANIMA/ANIMA_method/output/RData. This only needs to be done once, as the RData files persist on the user's filesystem after shutting down ANIMA.

9. The ANIMA GUI requires the ANIMA Neo4j database and several of the output files of ANIMA_build.R to function. Ensure that all output exists:

    a. Option 1: Use the precomputed output downloaded earlier (step 5, above)

        i. Shut down ANIMA: navigate to ~/ANIMA/ANIMA_method/compose_small and shut down all containers with: docker-compose down
        ii. Copy or move the compressed graph database (~/ANIMA/ANIMA_method/output/build/version_2017-07-20_18_47_11/graph.db.zip) to ~ANIMA/ANIMA_method/output/db and uncompress there. This replaces the existing graph.db folder, which was created the first time Neo4j was stared
    b. Option 2: re-compute all intermediate output and build the ANIMA database from scratch. This requires running Docker with at least 55 GB of RAM, and using the "compose_big" docker-compose.yml script. This may take up to 48 hours (!), depending on the system.
        i. From the RStudio interface, open Home/common/ANIMA_build.R and run or source the script. A lot of output (about 17 GB) will be produced in ~/ANIMA/ANIMA_method/output/build/version_XXX. Each time the script is run, a new output folder is created with the current date and time. The Neo4j database is created and populated on the fly and is complete when the script terminates successfully.

10. With the complete computational environment, source data, metadata, scripts, output and ANIMA database in place, we can start the ANIMA graphical user interface called ANIMA REGO.
    a. From the RStudio interface, open the file Home/common/ANIMA_Apps/ANIMA_GUI3_new.R

b. In the “Run App” dropdown, select “Run External”, and click “Run App”. A new browser tab will open entitled “ANIMA REGO method” at http://localhost:10787/p/3873/ . Starting up the Shiny App takes a while, and is complete when the module network for the *black* WGCNA module opens under the tabs “Single module” -> “d3graphSP”

c. With the interface up and running, refer to the ANIMA REGO manual (Supplementary pdf) for further information.

ANIMA REGO walk-through

When ANMA REGO is launched, a separate browser window or tab opens. The application is controlled via various control elements in the left pane, and output is viewed in the right pane.

Interface elements

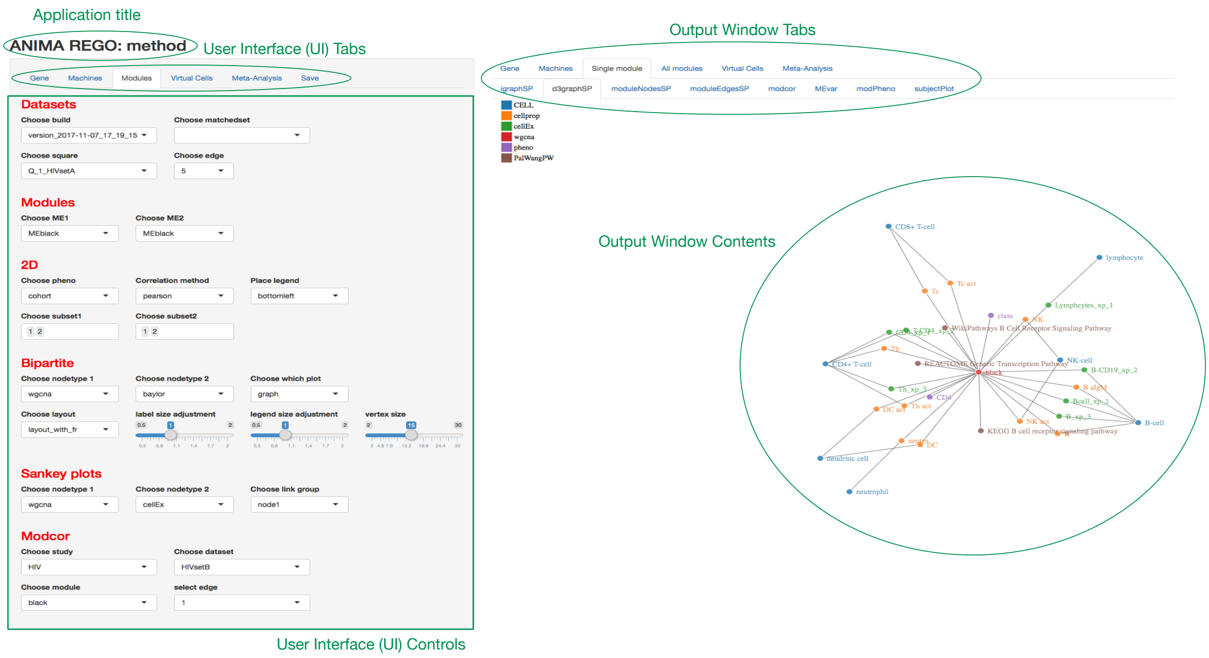

The application title displays ANIMA REGO and the name off the ANIMA project; in this case the project is called “method” as this project was developed for the paper describing the ANIMA method. There are six user interface tabs, each opening a page of various controls, described in detail below.

The output window is controlled by a set of nested tabs, which match up with corresponding user interface tabs. The largest area of the interface is reserved for output. In the sections that follow, we will explore all the tabs and their associated functionality.

Gene-level analysis

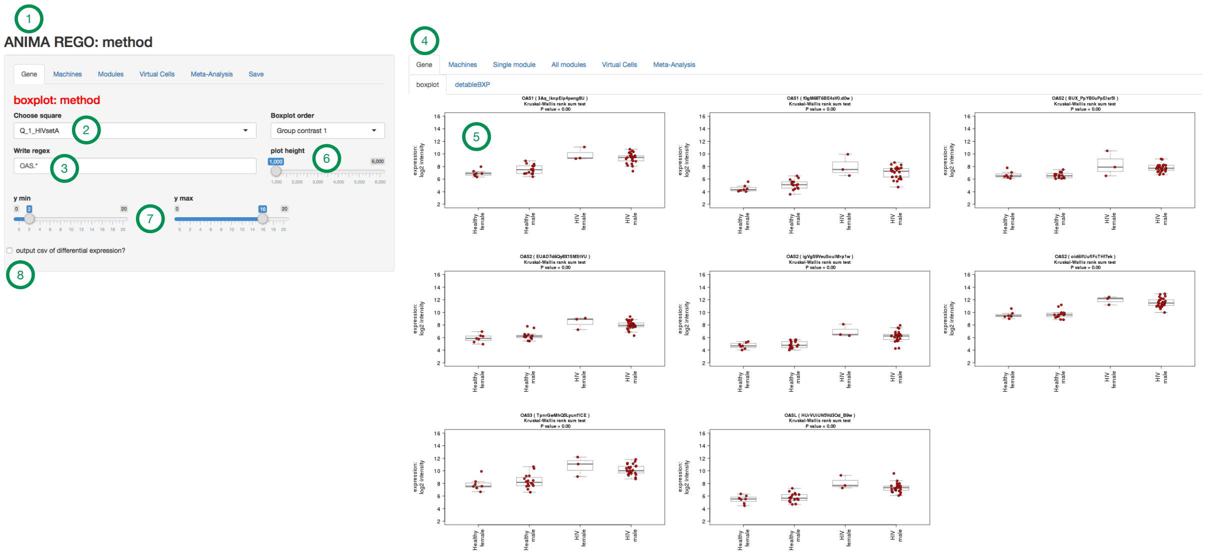

Probe- or gene-level expression data of individual or small groups of probes/ genes can be explored for individual datasets in the ANIMA database.

1. Click on the “gene” UI tab
2. Select the dataset
3. Type in a regular expression that represents one or more HGNC gene symbols (here we search for ‘OAS.*’, i.e. genes with names beginning with ‘OAS’
4. Click on the “Gene” output tab.
5. A series of box-and-whisker plots appear, with four categories each, showing the log2 intensity of the probes representing the search genes. To facilitate comparisons, boxplots can be grouped by either of the two factorial variables.
6. The height of the plot output can be varied depending on the number of plots shown
7. The minimum and maximum of the y-axis can be varied
8. A csv of tabulated results can be exported

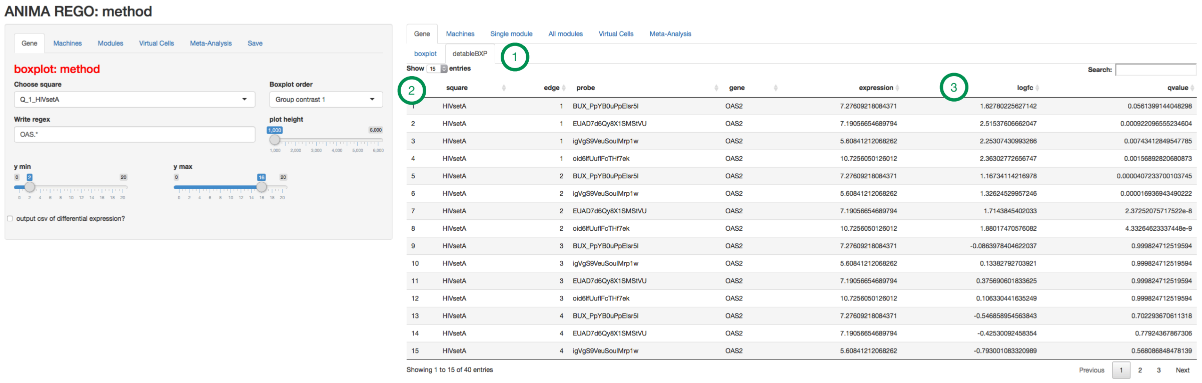

The graphical results presented in the “boxplot” tab can also be shown as a table

1. Click on the “detableBXP” tab
2. A table with various comparison statistics is shown, obtained from the Neo4j database
3. The table can be reordered based on the various headings, and is searchable

Molecular Machines: Inflammasomes

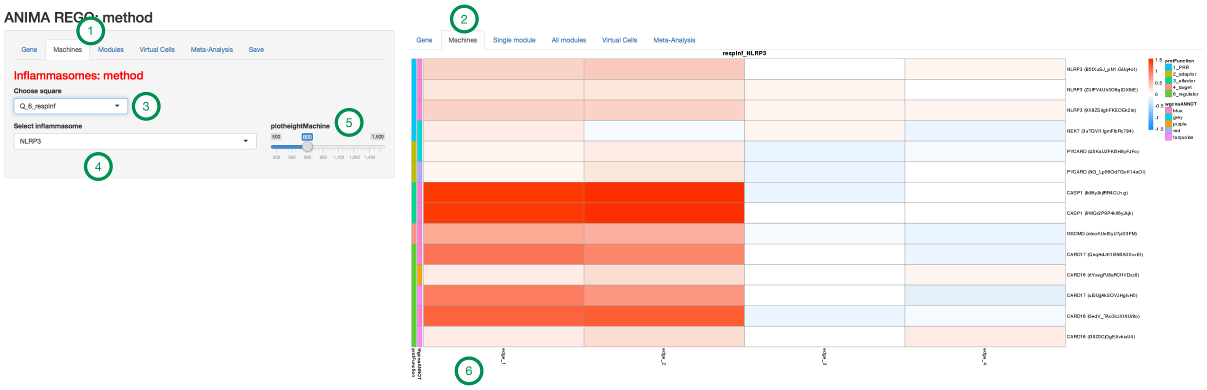

Relatively small numbers of gene products can act together as “molecular machines”. Here we look at inflammasomes, a family of protein complexes involved in intracellular information processing relevant to inflammatory processes. The “machines” included with ANIMA are illustrative; this functionality can easily be expanded.

1. Click on the “Machines” UI tab
2. Click on the “Machines” output tab
3. Select the dataset (in this case acute influenza)
4. Select the inflammasome of interest (NLRP3)
5. (This functionality is currently unavailable)
6. The output consists of a heatmap of log2-fold change values in expression of the probes representing the inflammasome genes. Here we show upregulation (or overabundance) of most of the inflammasome components in acute influenza (edges 1 and 2) with few, if any significant differences between men and women (edges 3 and 4). The legend illustrates protein function and WGCNA module membership.

Analysis of single WGCNA modules

Module associations

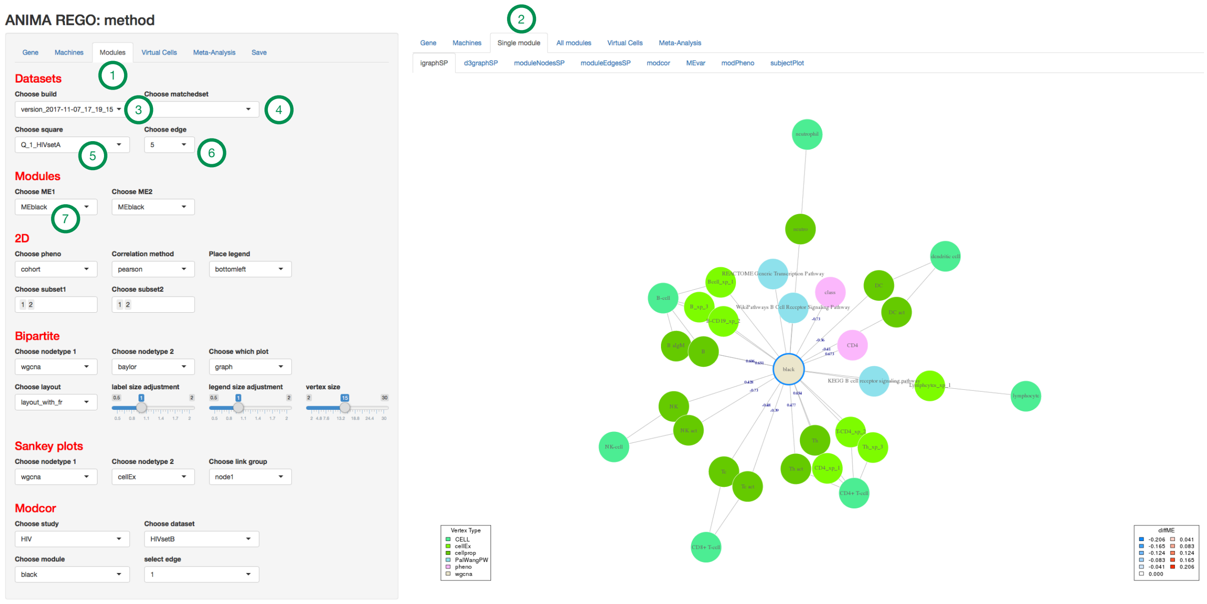

The cornerstone of analytic methods in ANIMA is weighted gene coexpression network analysis. The single module anlaysis tab addresses the problem of identifying the drivers and functional roles of individual WGCNA modules.

1. Click on the “Modules” UI tab
2. Click on the “Single module” output tab
3. Select the ANIMA build [non-functional, defaults to the latest build]
4. Choose matched set (only applies in datasets with paired samples, not applicable here)
5. Select dataset (Here we select HIVsetA)
6. Select edge (i.e the contrast). Here we select 5, indicating acute HIV vs healthy controls, regardless of sex.
7. Select a module (modules are named by colours). The output in the output window shows the module annotation network, i.e. the module and its associations with cell

types and pathways, extracted from the Neo4j database using a Cypher query and rendered using the *igraph* package. (All functionality implemented in a function called *igraph_plotter_newer.R*)

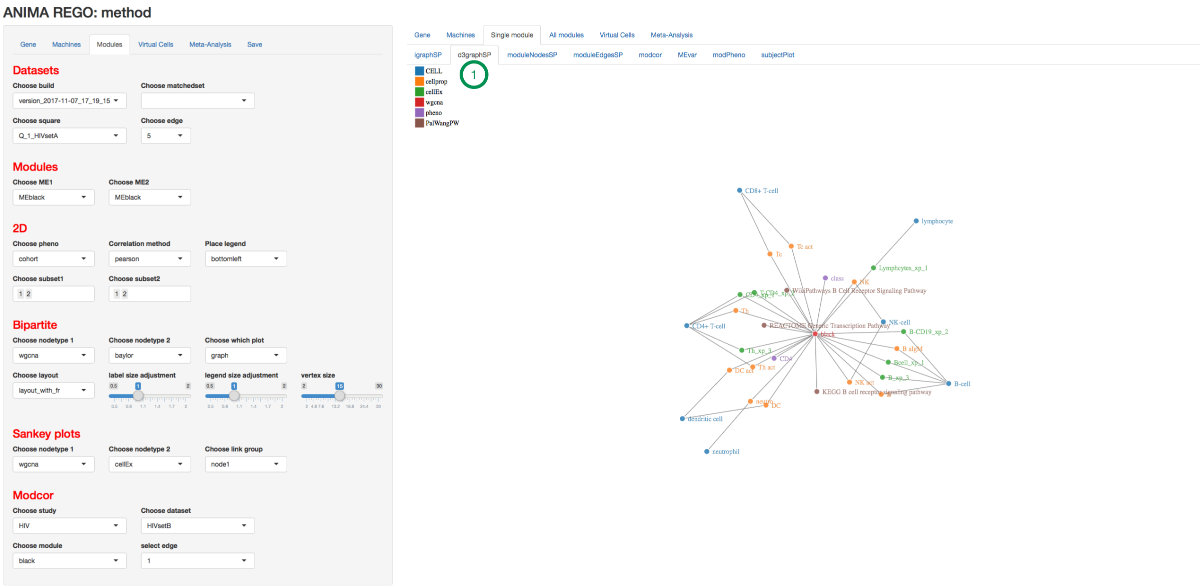

1. Click the “d3graphSP” output tab. The output now shown is the same networks as above, rendered using the *networkD3* library, based on the D3 javascript library

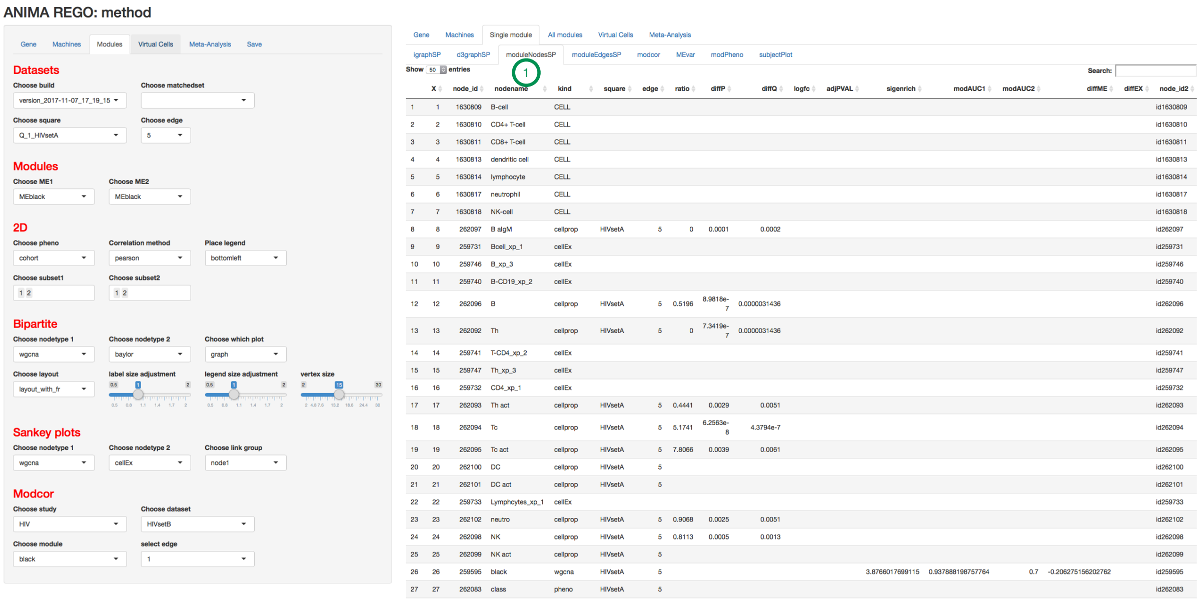

1. Click on the “moduleNodesSP” output tab. The output window now lists all the properties of the nodes in the graph(s) shown above. The table is scrollable, searchable and sortable.

ANIMA REGO: method

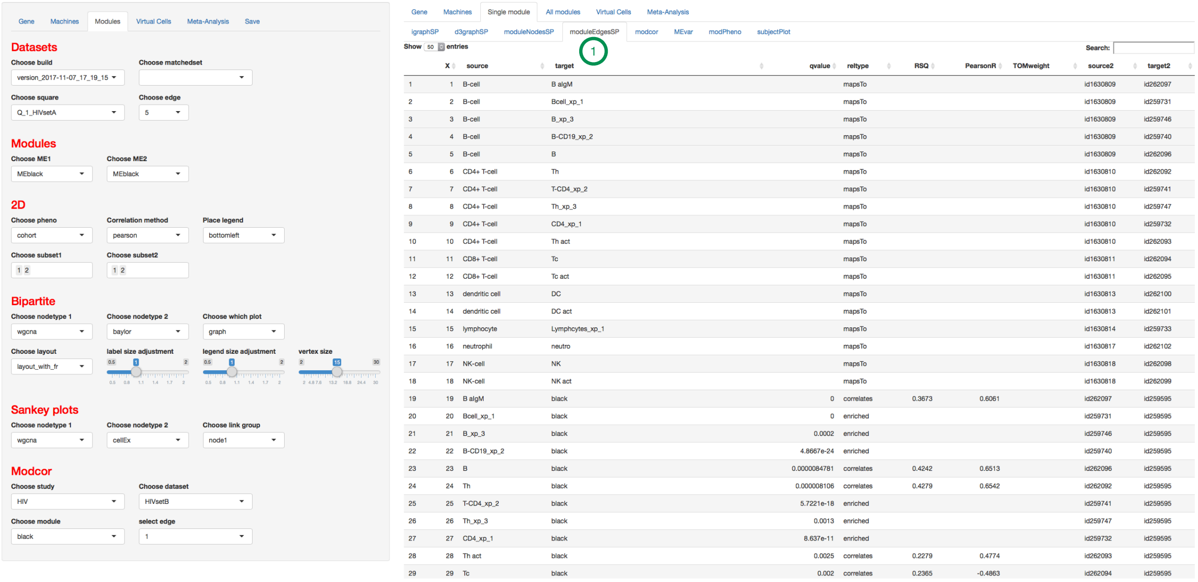

1. Click on the “moduleEdgesSP” output tab. The output window now lists all the properties of the edges in the graph(s) shown above. The table is scrollable, searchable and sortable.

Structural module analysis

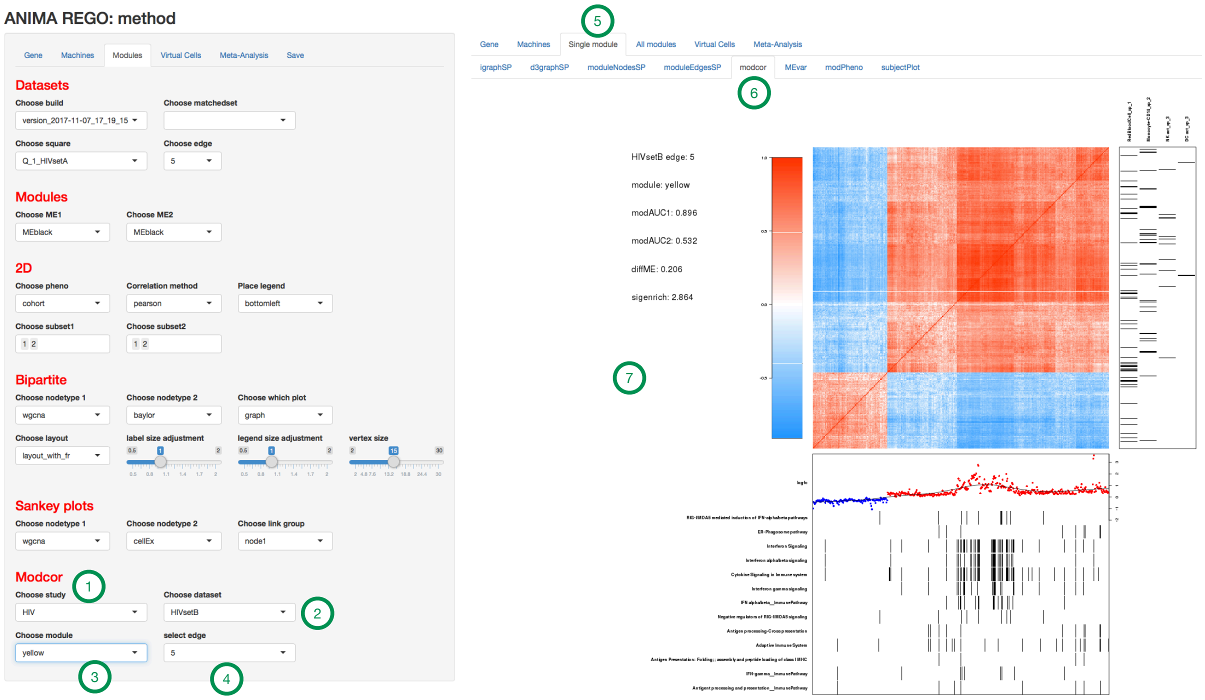

A WGCNA module is a group of co-expressed genes (as measured by probe intensities). Structural module analysis provides information on the correlation network underlying the module in a particular condition, as well as highlighting pathway and cellular context.

1. In the “Module” UI tab, choose the study from which the data is to be analysed (the study may contain more than one dataset)
2. Choose the dataset
3. Choose the module
4. Choose the contrast (“edge”)
5. Click the “Single module” output tab
6. Click the “modcor” tab
7. The output is presented as a clustered heatmap of the Pearson correlation matrix of the probe intensities of the selected module. This is annotated with two picket plots indicating probes mapping to biological pathways and cell-type specific gene lists.

Within the correlation matrix, patterns of tight correlation correspond to certain pathways. In addition, the log2-fold change of the probes in the module for the selected comparison (“edge”) is shown. Hence, we can get a good overview of *differential co-expression*, and relate this to both pathways and cell types.

Module eigengene variability

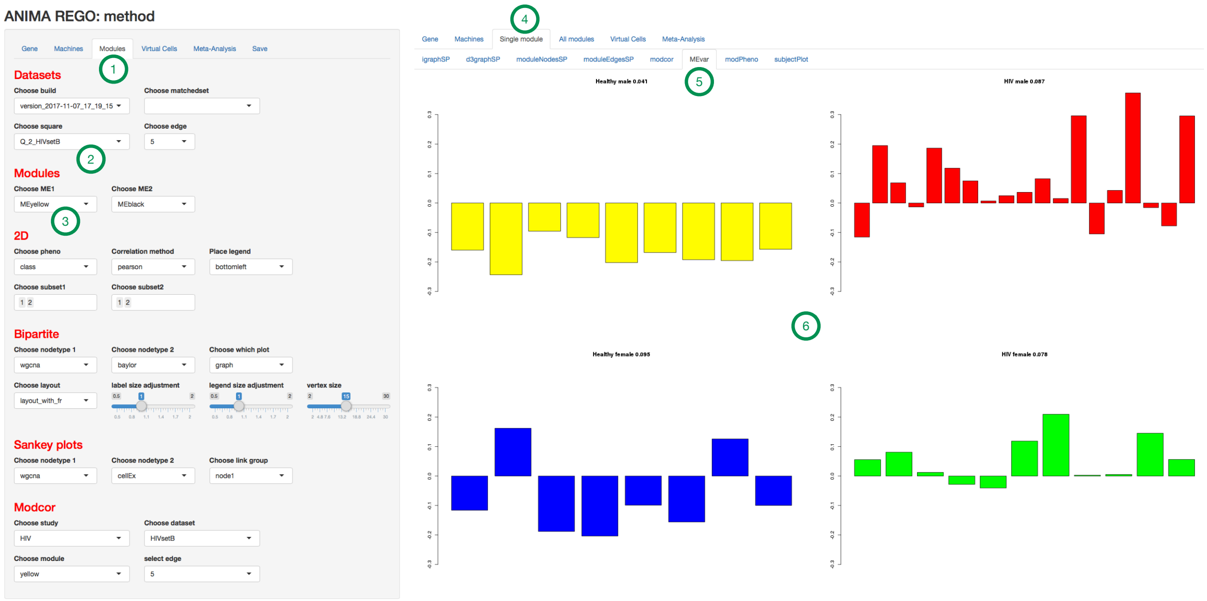

The module eigengene (ME) of a WGCNA module summarises the expression of the module on a per-sample basis. Here we visualize this ME broken down into the four sample groups defining the factorial "square".

1. Click on the "Module" UI tab
2. Select a dataset ("HIVSetB")
3. Select a module ("yellow")
4. Click on the "Single module" output tab
5. Click on the "MEvar" tab
6. The output indicates that, generally, the module expression is low in healthy samples (15/17 samples) and high in acute HIV (23/28) samples, suggesting that the modular expression profile may distinguish between acute HIV and healthy controls. The median absolute deviation for each of the four groups is shown above each plot, giving an estimate of intra-group heterogeneity of module expression.

Module eigengene correlation with clinical phenotype variables

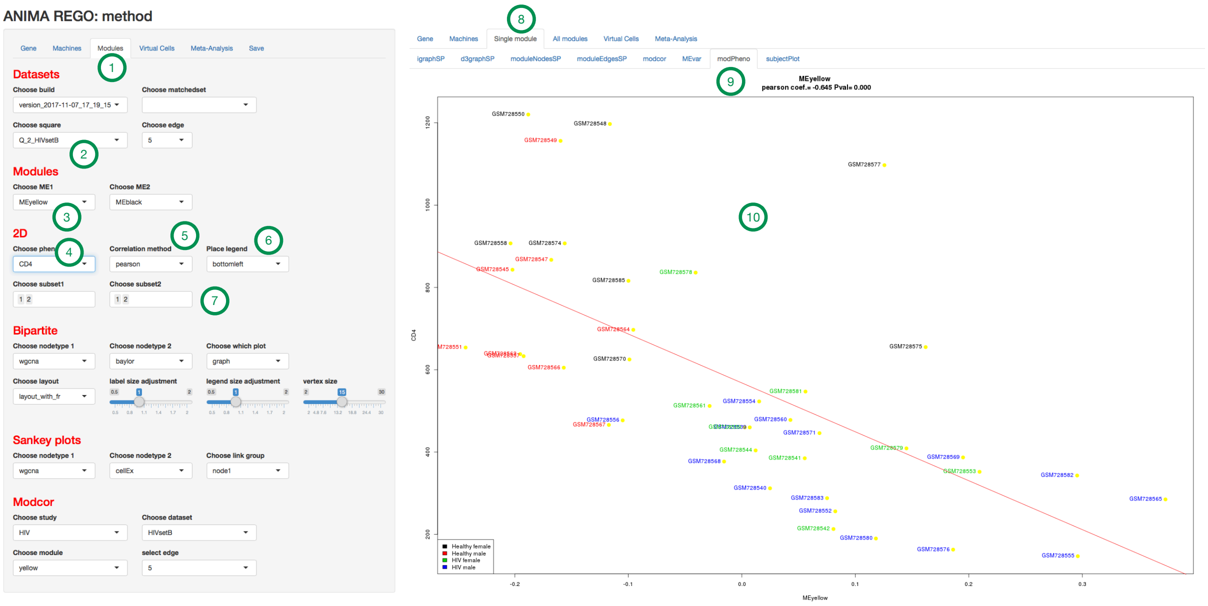

WGCNA module eigengenes can be correlated with clinical phenotype.

1. Click on the “Module” UI tab
2. Select a dataset (“HIVSetB”)
3. Select a module (“yellow”)
4. Select “CD4” from the “choose pheno” drop down
5. Select “pearson” from the “Correlation method” dropdown
6. Select “bottomleft” from the “Place legend” dropdown
7. Data subsets can be selected here, leave all selected
8. Click on the “Single module” output tab
9. Click on the “modPheno” output tab
10. The plot shows the *yellow* module eigengene vs CD4 count, demonstrating significant negative correlation (Pearson coefficient and P-value shown in the plot heading). Samples are coloured according to class

Subject plot

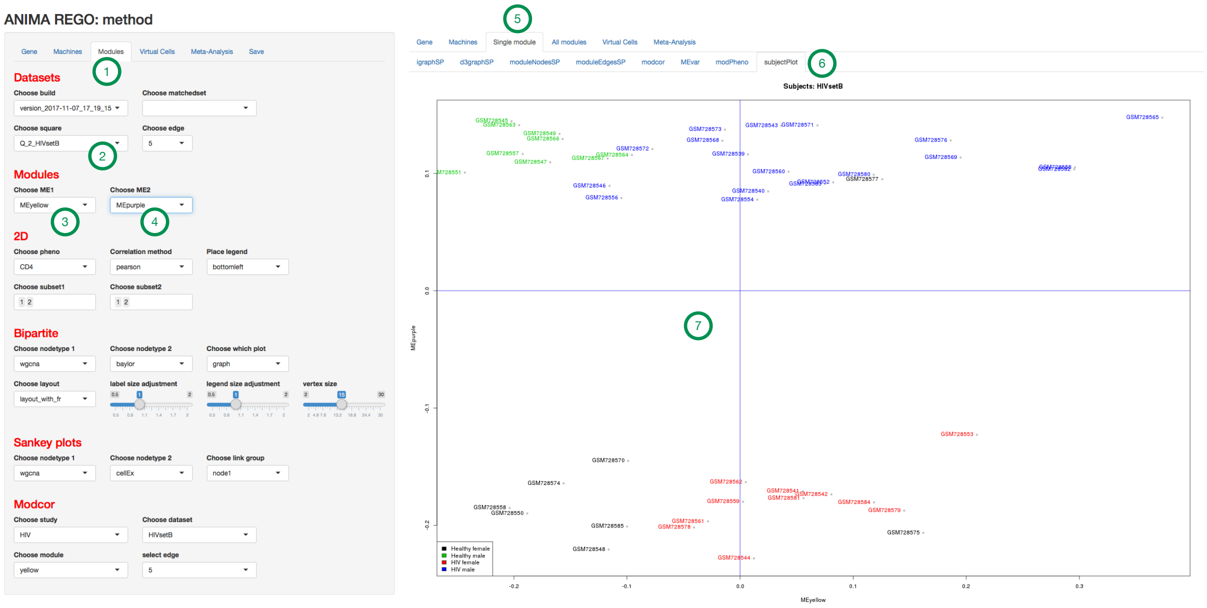

Two module eigengenes can be used to separate the samples into distinct groups.

1. Click on the “Module” UI tab
2. Select a dataset (“HIVSetB”)
3. Select a module (“yellow”)
4. Select a second module (“purple”)
5. Click on the “single module” output tab
6. Click on the “subjectPlot” output tab
7. Shown is a scatterplot of two module eigengenes. The *yellow* ME separates samples based on disease class, and the *purple* ME separates sample based on sex (with one misclassification, probably due to miscoding in the original clinical data).

Analysis of all WGCNA modules

Module 2D

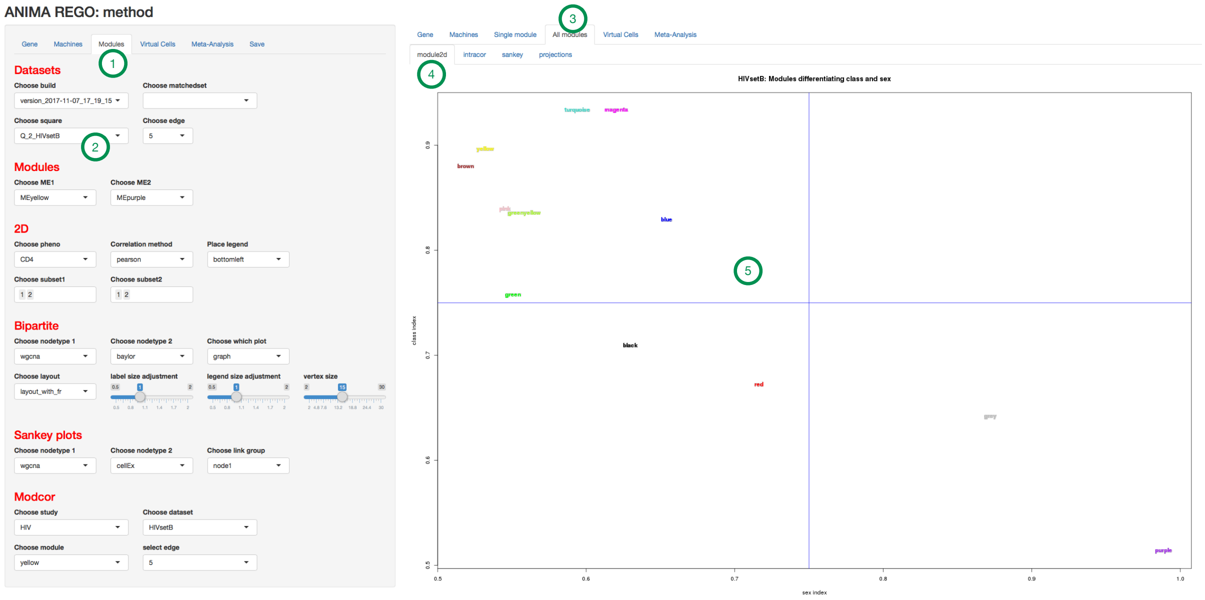

It is useful to obtain an overview of the modules that associate particularly strongly with either of the two factorial variables in the experimental design of the analysis. Here we show the example of acute HIV and sex in the HIVsetB dataset.

1. Click on the “Module” UI tab
2. Select a dataset (“HIVSetB”)
3. Click on the “All modules” output tab
4. Click on the “module2d” output tab
5. Shown is a scatterplot of all modules’ *class index* (i.e. **modAUC1**) vs *sex index* (i.e **modAUC2**) on a scale of 0.5 (no classification) to 1.0 (perfect classification). Clearly, individual modules differ in their strength of association with the two factorial variables.

Sankey plots

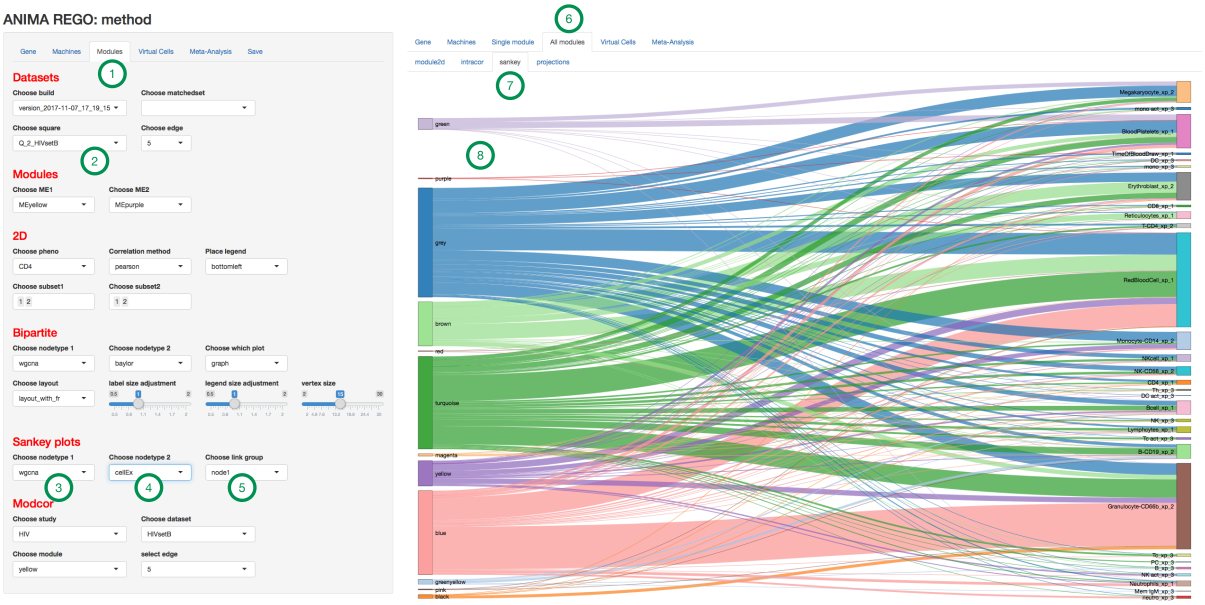

Another approach to visualize the relationship between WGCNA modules and other node types representing gene lists (e.g. lists of cell-type specific genes or biological pathways) is to use Sankey diagrams.

1. Click on the “Module” UI tab
2. Select a dataset (“HIVSetB”)
3. Select the first node type (“wgcna”)
4. Select the second node type (“cellEx”)
5. Select the link group, used to determine link colours (“node1”)
6. Select “All modules” output tab
7. Select “sankey” output tab
8. The plot shows the WGCNA modules on the left, with box size proportional to number of probes, and cell types on the right.

Bipartite graphs and their projections

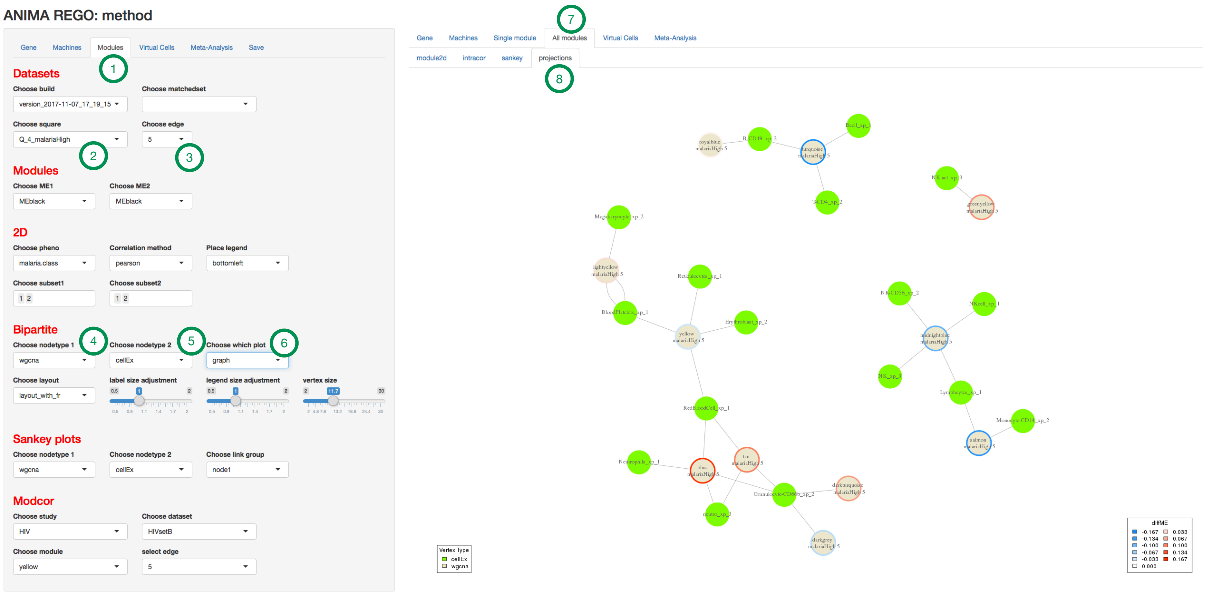

The Sankey plots above give and overall impression of modules linking to cell types. A more formal approach uses the hypergeometric test, and results in bipartite networks. Various of these can be visualized in the ANIMA REGO application.

1. Click on the "Module" UI tab
2. Select a dataset ("malariaHigh")
3. Select a contrast/ edge ("5", i.e. symptomatic malaria vs healthy controls)
4. Choose node type 1 ("wgcna")
5. Choose node type 2("cellEx")
6. Choose the plot ("graph", i.e. the bipartite network)
7. Click the "All modules" output tab
8. Click the "projections" output tab. The result is a bipartite network; edges represent significant association (hypergeometric test, P<0.05 after Benjamini Hochberg multiple testing correction). Rim colours of WGCNA nodes indicate over- or underexpression of the module in the particular comparison by visualizing the

“diffME” parameter as a coloured ring.

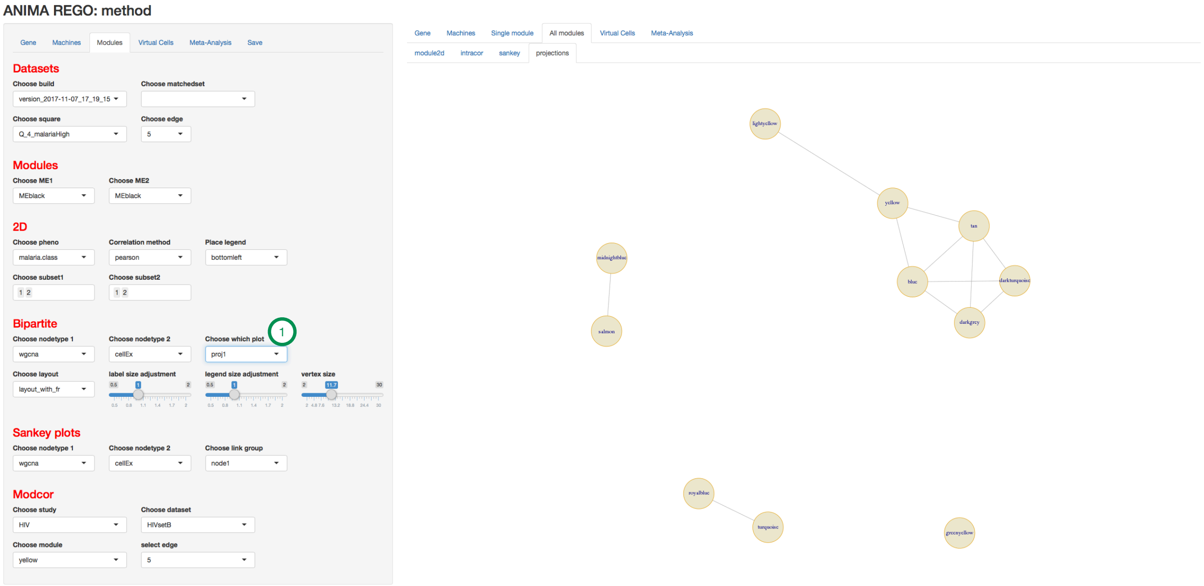

1. Now select “proj1” to select the first projection of the network. Shown are clusters of WGCNA nodes, that are associated because of shared cell type representation, suggesting a “myeloid” and “lymphoid” grouping.

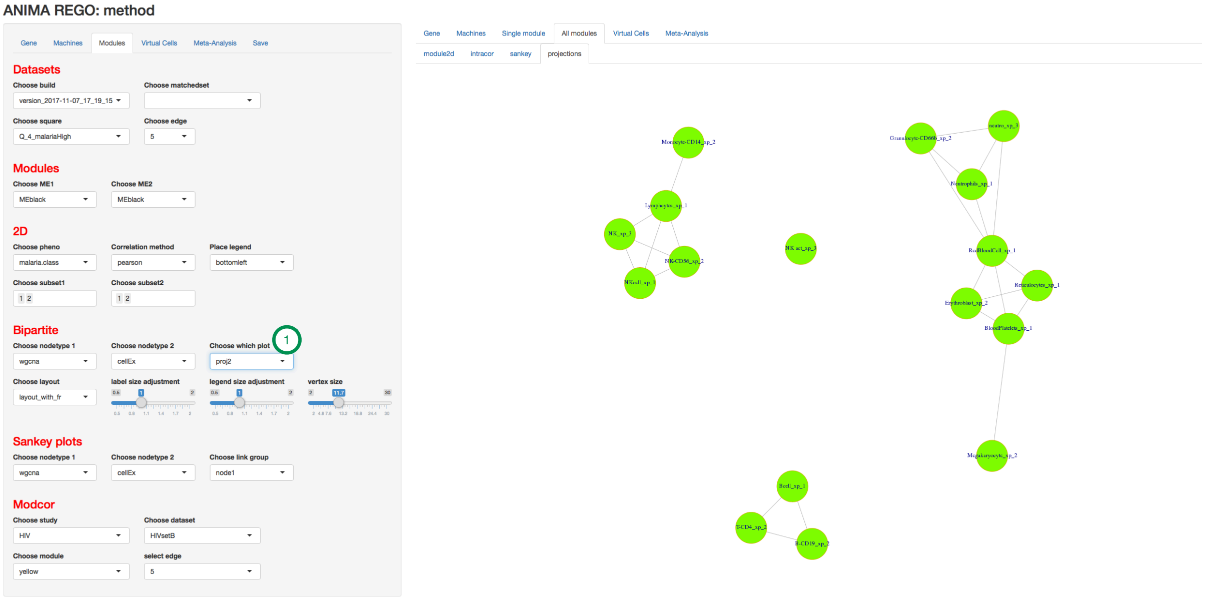

1. Now select “proj2”, the second projection of the bipartite network. Lymphoid and myeloid cells form distinct clusters.

Analysis at cellular level

Single virtual cells

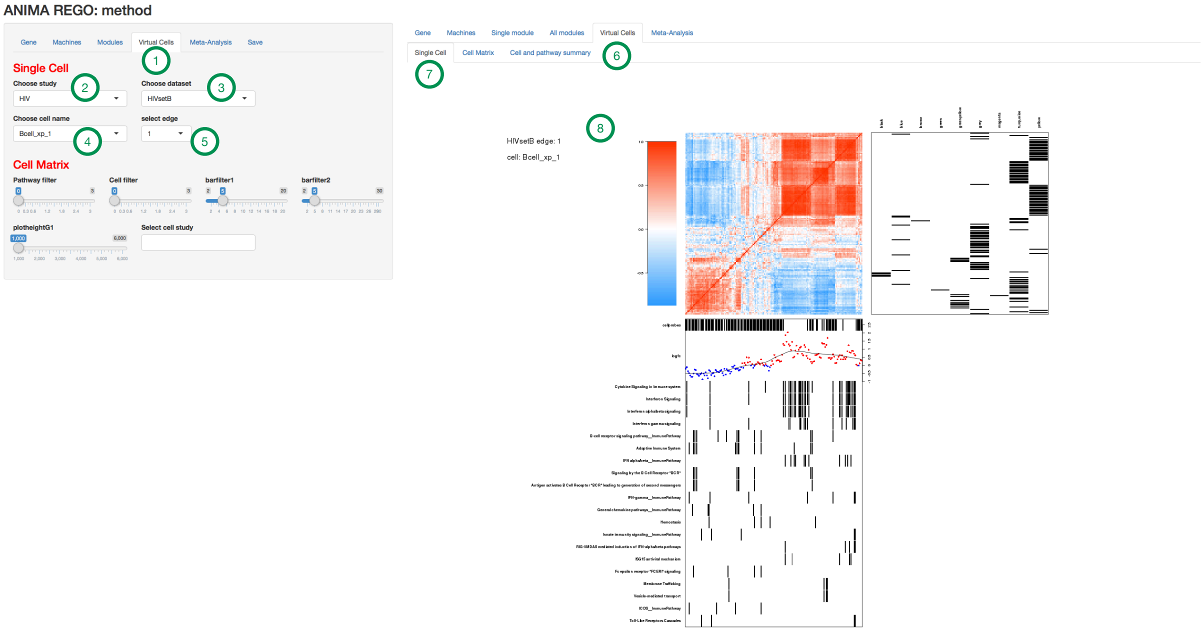

We now focus on virtual cells, introduced in the ANIMA manuscript. These are entities derived from cell-specific gene lists and genes whose expression is closely correlated with theirs.

1. Click on the “Virtual cells” UI tab
2. Choose the study (“HIV”)
3. Choose the dataset (“HIVsetB”)
4. Choose a cell to visualize. Take note of the suffix, indicating the origin of the cell-specific gene list.
5. Select the comparison/ edge (“1”)
6. Click on the “Virtual cells” output tab
7. Click on the “Single cell” output tab

8. The result is shown in the output window. The calculation may take a few seconds to a minute. Shown is a correlation matrix of probes constituting the virtual cell. This is annotated with pathways and log2-fold change values for individual probes. Also indicated are the “cell-type specific” probes used to infer the virtual cell, as well as module membership of the probes.

All virtual cells, one condition

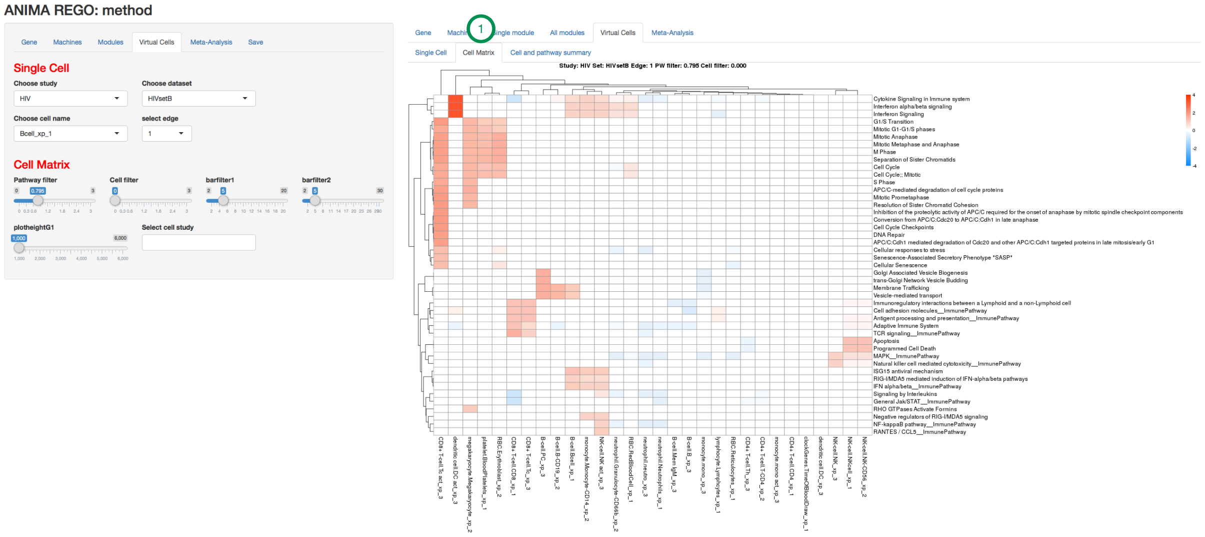

Zooming out, the focus now shifts to the behavior of all virtual cells

1. Click on the “Cell Matrix” output tab. Shown is a heatmap of cells and pathways, showing the “pathway activity scores” of all pathways in all cell types. To improve legibility, the plot is filtered, only including pathways with relatively high scores across the rows

ANIMA REGO: method

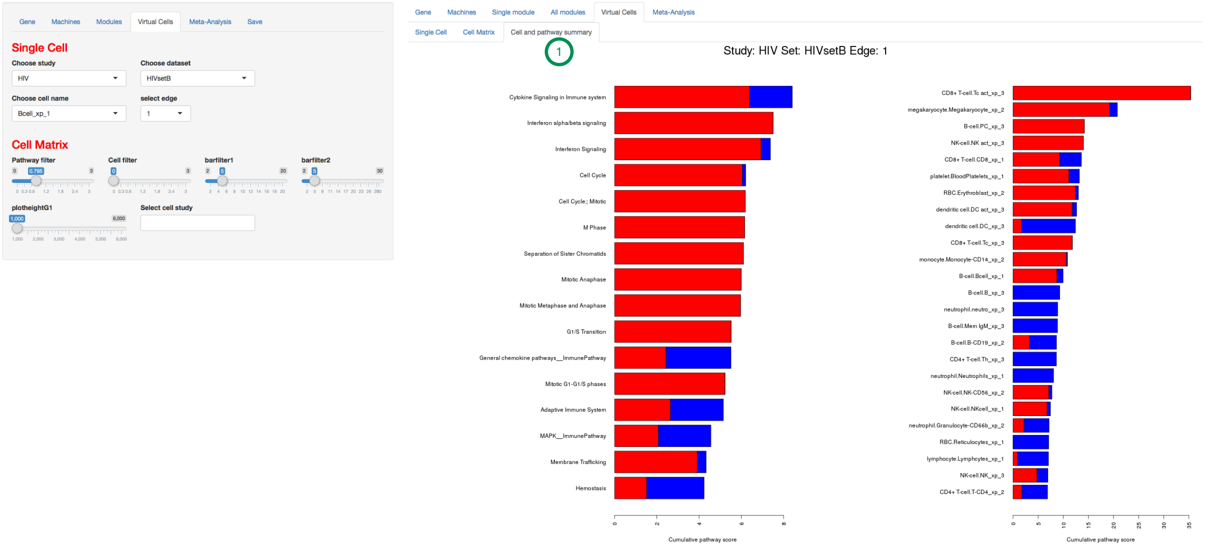

Zooming out further, the 2-dimensional heatmap is further reduced to two one-dimensional plots, consisting of the row sums and column sums of the heatmap

1. Click on “Cell and pathway summary” output tab. Now shown are cumulative pathway scores for pathways (left) and cell types (right), indicating the predominant biological processes and the key cellular players in the contrast of interest.

Meta-analysis

Module-level

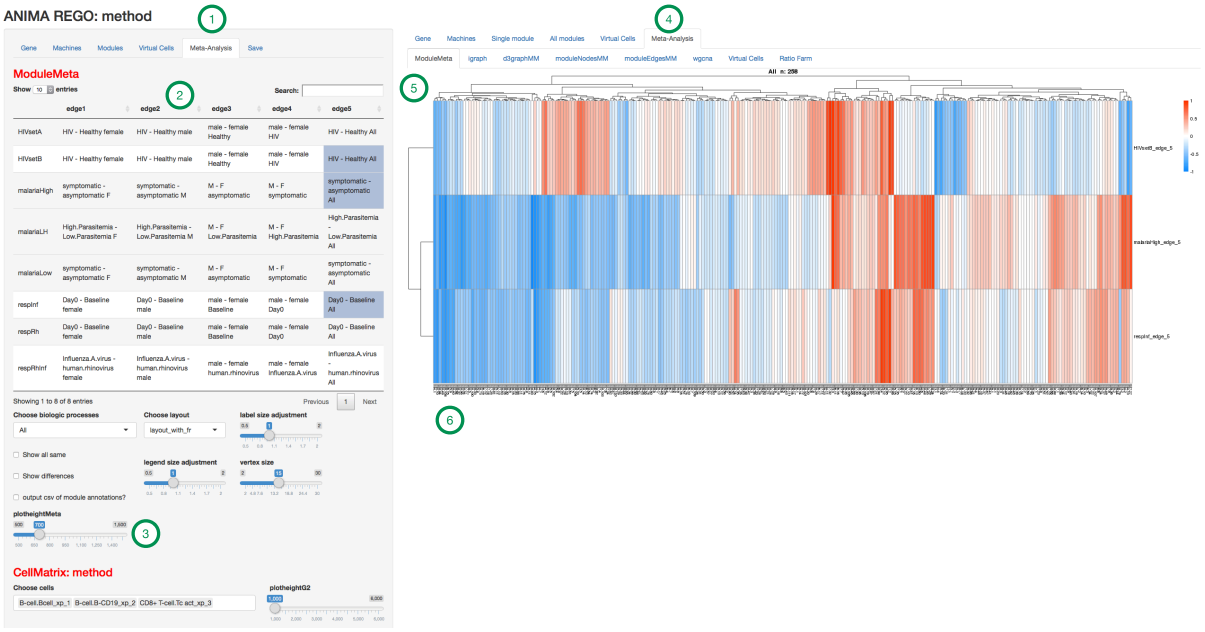

We now shift focus away from single conditions and datasets and determine similarities and differences between multiple datasets.

1. Click on the “Meta-Analysis” UI tab
2. Select an arbitrary number (at least two) conditions from one or more datasets. The leftmost column of the selection matrix lists the datasets, and the subsequent five columns display the contrasts/ edges of the factorial square. The text in the selectable fields clarifies the data subset and contrast. In this example, we compare acute HIV, symptomatic malaria (with high parasitaemia) and acute influenza infection, three rather different infectious diseases.
3. This slider controls the height of the plot
4. Select the “Meta-Analysis” output tab
5. Select the “ModuleMeta” output tab

6. The heatmap shows the difference in Chaussabel module expression (**diffEx**) for the relevant contrasts in all three selected datasets. Of interest is the relative similarity of responses to malaria and acute influenza, compared to acute HIV.

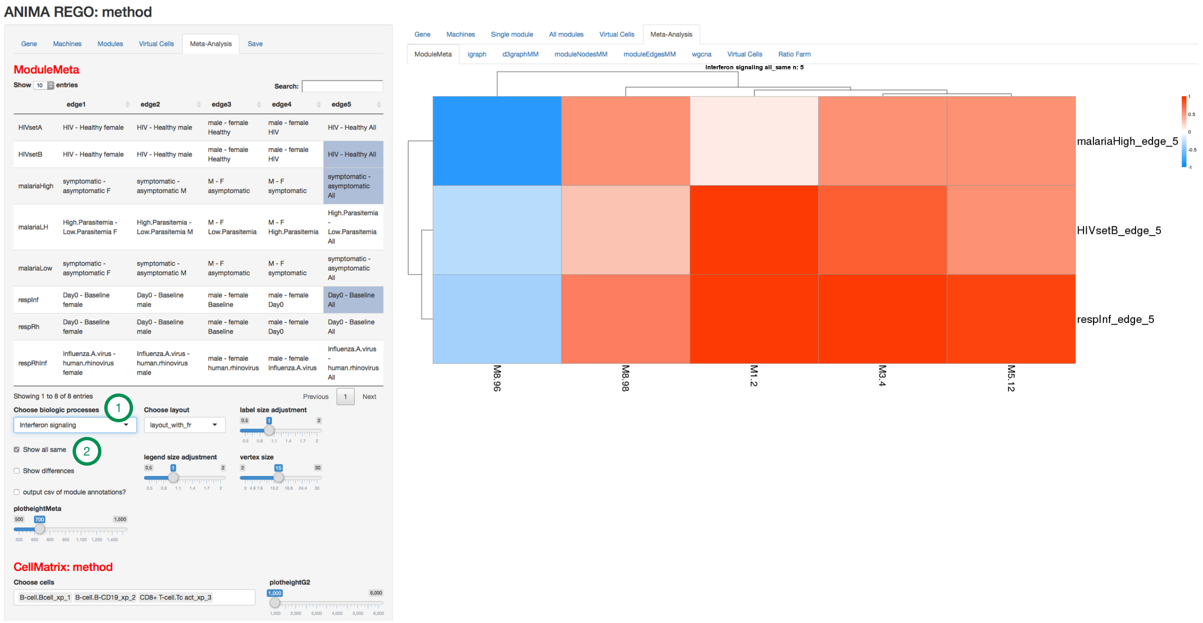

1. Now select “interferon signaling” in the biological process dropdown, and
2. Select the “show all same” checkbox. The visualization is now restricted to interferon-related modules with similar expression patterns across the three conditions, shown in the heatmap

ANIMA REGO: method

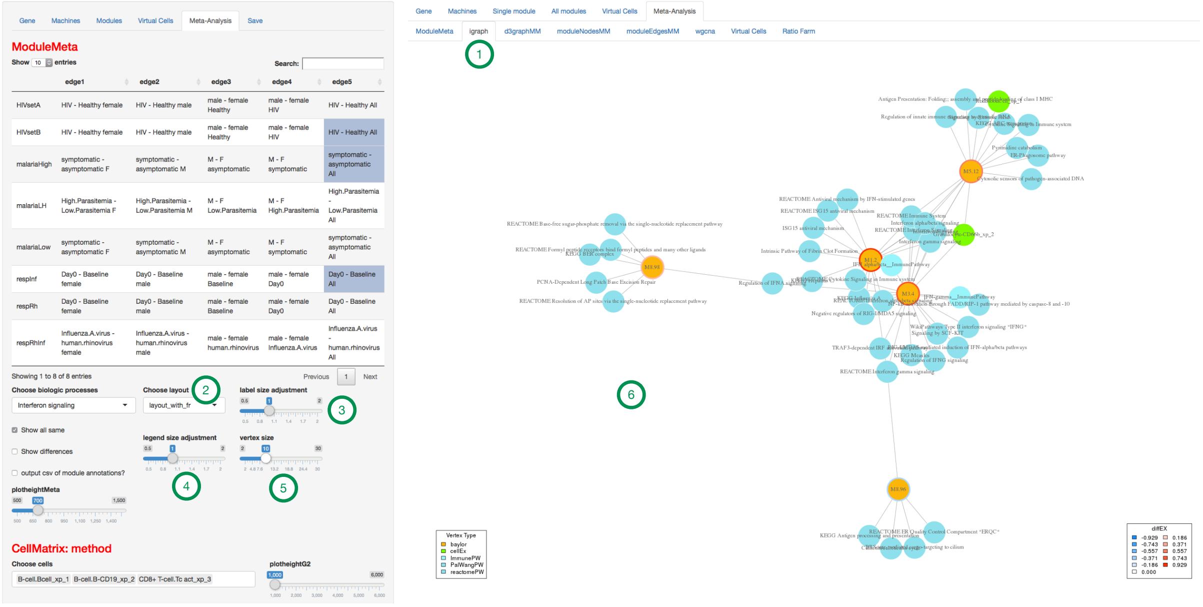

As it is of interest what processes and cells these modules relate to, we investigate the annotation graph (produced with *igraph_plotter_newer*)

1. Click on the “igraph” output tab
2. The graph layout can be selected here
3. Node label size can be adjusted for legibility
4. The size of the two legends can be adjusted
5. The size of the vertices can be adjusted

Caution: Only display the annotation network for a relatively small number of Chaussabel modules as the process will otherwise take a long time.

ANIMA REGO: method

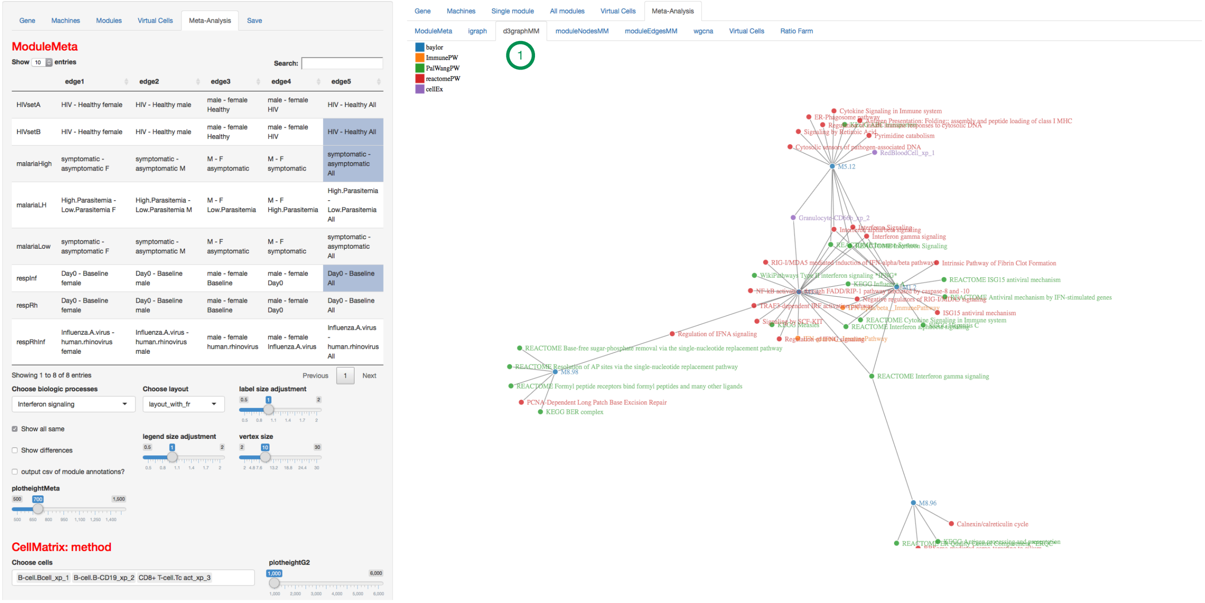

1. Now click on the “d3graphMM” tab. The same graph is shown using a D3-powered networkd3 function. The graph is zoomable, and nodes can be repositioned

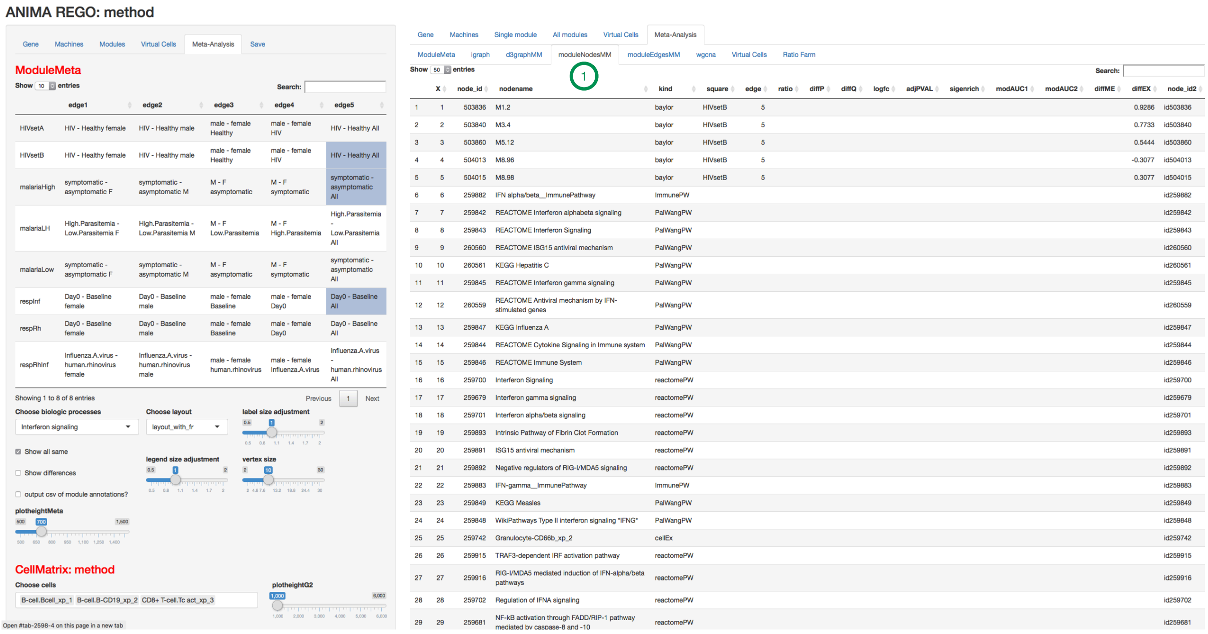

1. Now click on the “moduleNodesMM” output tab. Shown is a table of node properties for all the nodes in the previous networks

ANIMA REGO: method

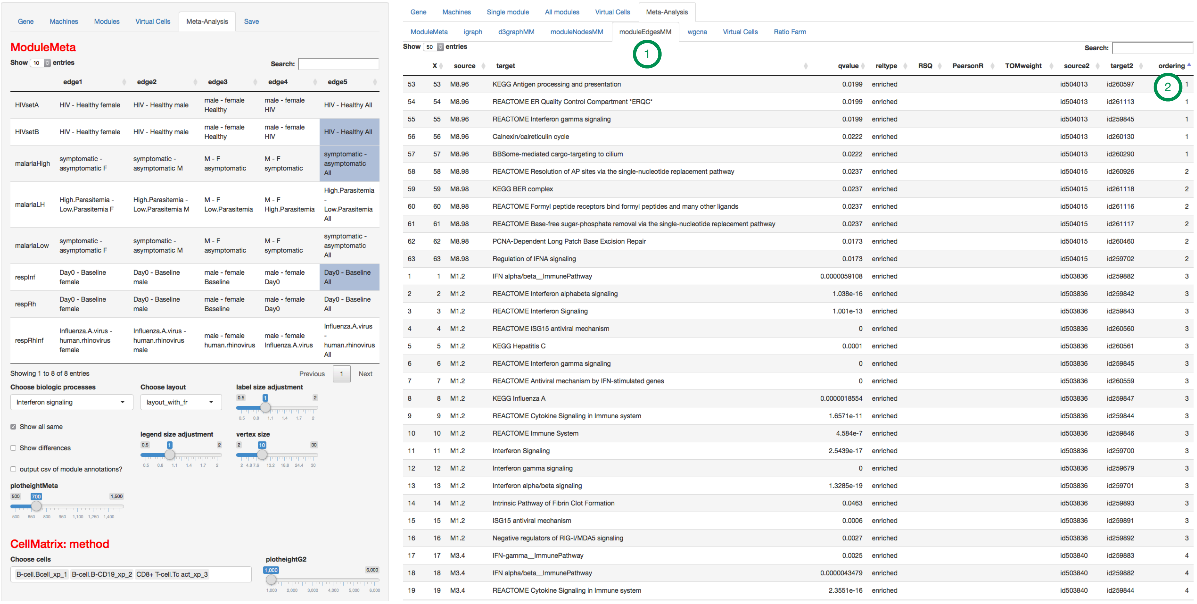

1. Now click on the “moduleEdgesMM” output tab. Shown is a table of edge properties for all the nodes in the previous networks
2. Importantly, sorting the column “ordering” in ascending order, reorders the table entries to correspond with the order of the clustered heatmap, read left to right. This greatly aids in interpretation of the heatmap.

Cell-level meta-analysis

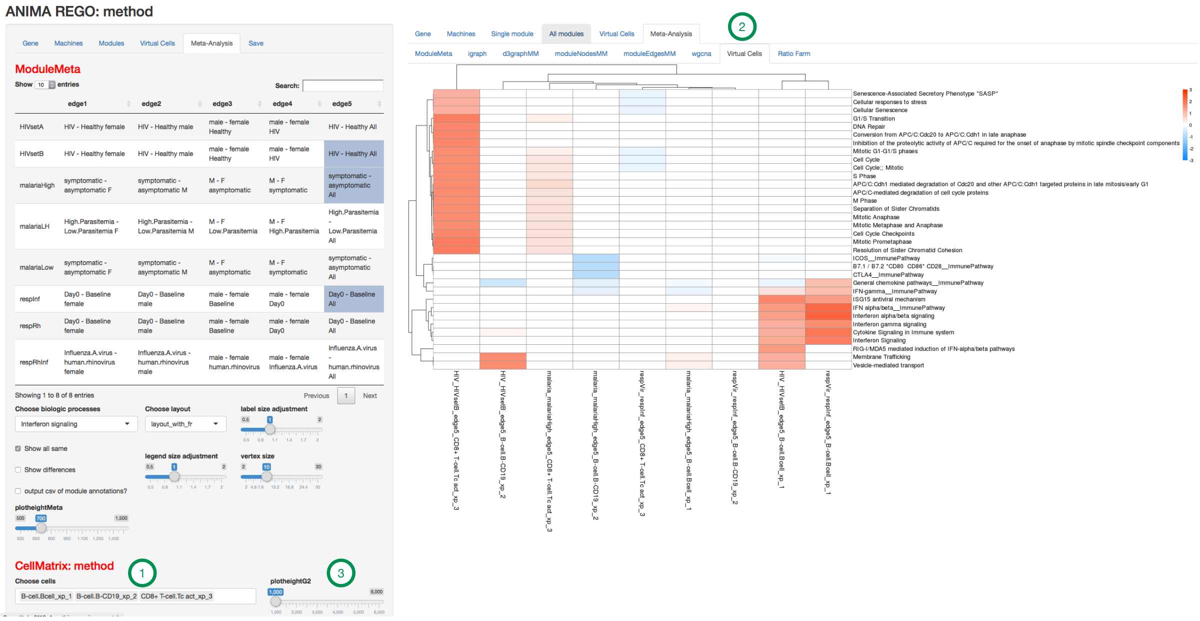

We now zoom out further and compare similarities and differences at the immune cell level across multiple studies. In this example, we focus on CD8+ T lymphocytes.

1. Select cell types
2. Click on the “Virtual Cell” Output tab. Shown are the pathways scores for various pathways in specified cells and specified studies, hence the long and complex labels.
3. The height of the plot can be controlled dynamically.
