# Supplementary material for: ANIMA: Association network integration for multiscale analysis
Source: Wellcome Open Res. 2018 Nov 14;3:27. Originally published 2018 Mar 12. [Version 3] doi: 10.12688/wellcomeopenres.14073.3 (PMC6134339; doi:10.12688/wellcomeopenres.14073.3)
Supplement: Peer review history

# Peer review: Referee response for version 3

**DOI:** 10.21956/wellcomeopenres.16261.r34252

**Published:** 2018-12-10

**Authors:** Emre Guney

**Affiliations:** 1 Research Programme on Biomedical Informatics, the Hospital del Mar Medical Research Institute, Pompeu Fabra University, Barcelona, Spain

The authors have appropriately addressed my previous comments.

I have read this submission. I believe that I have an appropriate level of expertise to confirm that it is of an acceptable scientific standard.

# Peer review: Referee response for version 2

**DOI:** 10.21956/wellcomeopenres.15932.r33740

**Published:** 2018-09-10

**Authors:** Emre Guney

**Affiliations:** 1 Research Programme on Biomedical Informatics, the Hospital del Mar Medical Research Institute, Pompeu Fabra University, Barcelona, Spain

Deffur and colleagues describes a transcriptomics data integration and analysis pipeline named ANIMA. ANIMA starts from raw microarray data sets, processes them using R and then applies WGCNA and another previously published module discovery method. The resulting networks (covering different relationships extracted from the data sets such as phenotype-differentially expressed genes, gene-gene co-expression, etc.) are stored using Neo4j graph database, allowing fast querying of relationships that satisfy certain criteria. In addition to the pipeline, the authors build a web application using Shiny. They also showcase the use of the pipeline by analyzing three publicly available data sets. The article explains the technical details of the pipeline clearly. Although ANIMA currently supports only Illumina BeadArray other platforms such as Affymetrix and sequencing data is planned to included in the future. Indeed, addition of other platforms and RNAseq data analysis capabilities would be a important improvement.

1. The intro falls short on existing related work, several recent efforts aim to integrate, analyze and store omics data as well as provide a reproducible work flow such as
   
  - Omics Integrator (Tuncbag et al., 2016, Plos Comp Bio)
  - NDEx (Pratt et al., 2015, Cell Systems)
  - nextflow (Di Tomasso et al., 2017, Nat Biotech)
2. How does ANIMA differs from GeNNet?
3. Are there any plans for including protein interaction network analysis tools such as BIANA (Garcia-Garcia et al., 2010, BMC Bioinformatics) into the pipeline? Also, how easy is to customize the ANIMA, say how easy to choose another co-expression network analysis software?
4. What do the P-values on the title of the Figure 3 correspond to? Which groups were compared?
5. I find using color names to represent modules in Figure 4 highly confusing, especially given that blue is also used to denote underexpression. They can be renamed and/or described better in the legend.

Minor:

- The focus on immune system in the beginning of the intro constitutes a slight disconnection with respect to the abstract / rest of the text. I understand the focus due to the case studies but still reads isolated from the main theme of the paper, which is introducing ANIMA as a framework. The authors might as well redirect the focus to inference of cell state for immune response but then they would need to provide a more stringent validation of the obtained results in terms of the biological findings.
- The use of subsections in the introduction feels rather unconventional for scientific writing and somewhat confusing given that the current approaches only mention one existing approach followed by the proposed approach.
- Question 2 in problem definition fails to pose a general question, going to the technical details before having explained the graph under concern, the triples, P-value correction etc.
- In fact, to me it seems that the authors describe the problem in the latter “rationale” sections, where they highlight the problem on the storage of research-related results and network data as well as the difficulty of reproducibility due to software / platform version changes.
- “We prefer scripted workflows ...” We? Scientists?
- “high order, high complexity and low dimensional state space vectors from high dimensional low order, low complexity data (i.e. non-normalised microarray data and clinical/sample phenotype” not clear what the authors mean by the order and complexity of the data (I see later in the figure 1 that it is biological complexity). Moreover, this sentence is very long and can use a revision.
- Some references are not in right order (e.g., Chaussabel refered as 14 while being 17).
- suffixes => suffices

I have read this submission. I believe that I have an appropriate level of expertise to confirm that it is of an acceptable scientific standard, however I have significant reservations, as outlined above.

# Response

**Published:** 2018-10-30

**Authors:** Armin Deffur

**Affiliations:** University of Cape Town, South Africa

Many thanks for the thorough review and comments. 

The introduction has been reworked to address most of the comments made in the referee report. It now reads more clearly. Suggested references have been incorporated. 

With respect to comment (4), the legend of figure 3 addresses the question. The introduction now reduces the focus on the immune system, first discussing the ideas underlying the ANIMA framework, before showcasing ANIMA by example data derived from immune responses to infectious diseases. 

Subsections in the introduction have been removed.

Vague allusions to the theory of complex systems have been either removed or made clearer. 

While I agree that colour names for WGCNA modules can be confusing, I avoided relabelling the modules manually to remain true to the original output. Replacing colours with numbers would not offer a significant benefit, and coming up with function-related names would be to some extent subjective. Indeed, the annotation of the WGCNA modules suggests that assigning singular descriptions to them is not straightforward.

# Peer review: Referee response for version 2

**DOI:** 10.21956/wellcomeopenres.15932.r33262

**Published:** 2018-06-26

**Authors:** Christopher L. Plaisier

**Affiliations:** 1 Arizona State University, Tempe, AZ, USA

Overall the revisions have improved the work substantially. The addition of Table 3 was helpful, but it left additional questions.

 Critiques:

1. Table 3 is helpful but the data types are named in ways that are not described anywhere in the main text. For instance baylor is Chaussabel modules. But it takes a lot of effort to figure this out. It would be very helpful to have an additional table that describes each data type. Without this the information in Table 3 is less useful.
2. The data type baylor in table 3 ought to be changed to Chaussabel for the sake of consistency.

I have read this submission. I believe that I have an appropriate level of expertise to confirm that it is of an acceptable scientific standard.

# Response

**Published:** 2018-10-30

**Authors:** Armin Deffur

**Affiliations:** University of Cape Town, South Africa

Many thanks for reviewing the second version of the paper.

Table 3 has been updated to indicate the "Chaussabel modules" are equivalent to the "baylor" datatype. In future versions of ANIMA we will change the datatype in the source code; as an aside it would be ideal to avoid eponyms for this, as in the case of WGCNA.

We did not include a table describing each individual datatype in the main text, but it is available in the Supplementary information as Table S5, and detailed descriptions are given on page 9-10 of the Supplementary file.

# Peer review: Referee response for version 1

**DOI:** 10.21956/wellcomeopenres.15308.r32374

**Published:** 2018-04-17

**Authors:** James A. Eddy

**Affiliations:** 1 Sage Bionetworks, Seattle, WA, USA

Deffur et al. present ANIMA, a network-based method for integrating gene expression (microarray), annotation data, and clinical data. ANIMA utilizes a merged graph database to represent and allow exploration of associations/relationships between biological processes and cellular contexts at multiple scales. The authors provide software (R, Docker) and extensive documentation for building and interacting with ANIMA networks for several example infectious disease datasets; they also present a web interface (R/Shiny application) for interactive visualization of results. The example datasets are used to demonstrate three central approaches for interpreting data with ANIMA, highlighting selected results and features of the method and tools.

 I would be interested in using this method for some of my own projects related to systems immunology and immuno-oncology, and the web tool seems like it would provide a nice tool for biologists who wish to explore a particular dataset. As a “framework,” ANIMA would be even more appealing to computational biologists as a more modular/extensible system — but still offers a healthy breadth of analyses in its current state. While this manuscript is technically sound, there are issues with structure and organization that I would recommend addressing. In particular, the authors should (1) more clearly describe the rationale for their methods; and (2) present method details more prominently in the main body of the manuscript.

**Major comments/critiques**

*Regarding rationale:*

1. The authors mention the ability to “discuss [immune response] not in the language of probes, genes and signatures, but rather as coordinated biological processes and the cellular context for these processes.” I like this idea, and I feel that it’s a key distinction of the ANIMA method. However, the authors should explain why they feel this approach is better or complementary to other, more feature-based multi-scale association networks (e.g., Vaske et al., Bioinformatics. 2010. 26(12)).
2. The authors do not indicate whether any similar methods to their own (e.g., Costa et al., PeerJ. 2017. 5:e3509 — perhaps) exist or, if so, describe how ANIMA compares to or improves upon such methods.
3. The authors should provide at least some justification for the technical details of their method and tools: Why use a graph database; why neo4j? How did they select specific packages/implementations for analytic approaches (Table 1)? Alternatively, the authors could demonstrate that associations in the final network are “robust” to choices of packages and parameters for individual analyses — however, this might be more appropriate for a follow-up study.

*Regarding method details:*

1. Given that ANIMA, the subject of this Methods article, is described as “a network-based data integration method,” the authors should present more details about the network building process — beyond the relatively brief overview in the “Method outline” section. The Supplementary Methods and Figures are immensely helpful for understanding the manuscript; suggestions for pieces of information to move or at least duplicate in the main text include:
  - Enumeration of full set of bipartite graphs (i.e., from Supplementary Methods ~ “Bipartite graphs based on association indices”, Figure S7) — a condensed version of the Supp. Methods section in table form would likely be sufficient; however, references to Figure 1 should clearly indicate that this figure is illustrative and does not include the full scope of output types and relationships used.
  - Reference to detailed methods for analytic approaches (i.e., from Supplementary Methods) — this could be included as an additional column in Table 2, referencing a specific page or section number in the Supplementary Methods. Additionally, it would be helpful to list the bipartite graphs for which each analytic approach serves as input or output (referencing the graph number 1-29 and/or the suggested table above).
2. Somewhere in the methods section, it would also be helpful to include a brief overview of how a user would approach and use ANIMA to build a new network for their data:
  - This point might be more obvious to some, but was lost on me at first: clarify that ANIMA networks produced by the method is specific to the input dataset(s), and there’s not a central, global “ANIMA” graph database (though providing a repository to share and explore user-generated ANIMA networks could be an exciting future direction).
  - A simplified, more process-oriented version of Figure S9 (don’t need to list specific functions and scripts, but diagram and summary of inputs, outputs, and steps) would be great.
  - Describe computational requirements (operating system, CPUs, RAM, time, etc.) to perform various steps.
  - Describe which microarray platforms are supported and any expected format requirements for the input data.
3. The three approaches presented in the “Results” section give helpful examples of how a user can interact with an ANIMA network, once constructed, and what can be learned from the method*. Additional results of interest include:
  - Performance (related to requirements, but actual runtime statistics for examples presented);
  - Comparison to other methods (if applicable).

*Note: I really think the authors could consider presenting the Shiny-based web tool (and corresponding “use cases”) as a separate “Software Tool” article, as the target audience and key results are subtly different than the ANIMA method; however, I leave this to the discretion of the authors.

**Minor/discretionary comments**

1. For the Cypher query example, a corresponding “translation” of the syntax would be helpful — either as something more SQL-like or a verbose, literal explanation of what the query is doing.
2. There’s a typo in Figure S10 (“F[i]lesystem”).
3. In the “Discussion” section, the reference to “selection of one or a few cell types” as a limitation of single-cell RNAseq is increasingly less true with technologies like 10x — might want to revise.
4. For differential transcript abundance prior to WGCNA, genes are “ranked” and the top 4000 are selected; similarly, the 500 are used in pathway enrichment. For Chaussabel modules, was any cutoff used to determine which genes were up- or down-regulated?
5. Authors should clarify that for this particular iteration (due to the choices of cell types from Abbas et al. and the Chaussabel modules), the method is most applicable to datasets with an immune component or context.

I have read this submission. I believe that I have an appropriate level of expertise to confirm that it is of an acceptable scientific standard, however I have significant reservations, as outlined above.

# Response

**Published:** 2018-05-20

**Authors:** Armin Deffur

**Affiliations:** University of Cape Town, South Africa

Many thanks for the thorough and comprehensive review. The following changes were made in order to address the major concerns:

1. We discuss the key distinction of ANIMA compared to other methods as the ability to generate virtual cells from the expression data and annotation libraries; the specific example referenced (PARADIGM) outputs rankings of pathways 
2. We thank the reviewer for pointing out the Costa et al paper, which was published after our own development of ANIMA. While both approaches rely on scripted workflows, graph databases and reproducible computational environments, our approach is sufficiently novel in its ability to generate new information from the multipartite graph.
3. A more detailed introduction is now provided, providing extended justification for the approaches used
4. The Methods section was reworked by moving some material from the supplementary information to the main manuscript, and adding a new Table 3, which lists the individual bipartite graphs. Figure S7 is now cross-referenced to Table 1, and the various workflow components indicated in the Table and Figure.
5. A section has been added for the requirements (hardware, data format, etc.) of using ANIMA for a user's own data, and the outline for the procedure is listed.
6. A simpler version of Figure S9 has been added as a subfigure to Figure 1 in the main manuscript.
7. We feel that the Shiny web application is key to delivering the functionality of ANIMA from a user perspective, and have retained the relevant sections. In future, once technical issues regarding hosting a multi-container based web-app have been solved, and we start hosting the web interface, we may publish an application note describing this. 
8. We have translated the Cypher syntax for the example query as suggested.
9. The typo in Figure S10 has been corrected.
10. The discussion of single-cell RNAseq has been revised.
11. The information on the cutoff for significant up- down regulation of genes in Chaussabel modules has been added to the relevant section in Supplementary information.
12. We emphasise the immunology use case for ANIMA in its present form, but leave open the possibility for additional use cases with appropriate code changes (future work section)

# Peer review: Referee response for version 1

**DOI:** 10.21956/wellcomeopenres.15308.r31798

**Published:** 2018-03-27

**Authors:** Christopher L. Plaisier

**Affiliations:** 1 Arizona State University, Tempe, AZ, USA

This manuscript provides a description of a method to infer many useful computational relationships from patient data in one comprehensive resource. The method is tested in proof-of-principle studies of human patient data from infectious diseases. The examples are fairly thorough, but cannot possibly cover the complex nature of the relationships that are potentially computed in a cohesive manner. Nor are the statistical filters that should be or are applied described in detail.

 Major critiques:

1. All the potential cutoffs and alpha values should be described and how to correct for multiple hypothesis corrections for each analysis, and overall. One good place for this might be Table 2.
2. A better table must be provided with the following column headers: "Data Type 1", "Data Type 2", "Statistical Method", "Implementation", "Biological Implication", "Description", "Reference(s)"
3. Figure 1a why is there a distinction between Biological Pathway and Chaussabel Module? They are both forms of prior information that is overlaid onto the network via the same method. A more accurate description would be "Gene Sets from Prior Information" or simply "Gene Sets".
4. The first section of the results is Validation study. This would be better described as an Example study as validation means studies done to confirm prior observations.
5. This paper reads more like a manual than a paper. One thing that would be very helpful would be a demonstration of how the network-based approach provides a synthesis of information and describes something novel or at least puts together things that weren't previously associated.
6. From a computational perspective, the motivation is clear. But from the perspective of a potential biologist why would they use this over something else.
7. What tools does this compare to and how is your method an improvement?

I have read this submission. I believe that I have an appropriate level of expertise to confirm that it is of an acceptable scientific standard, however I have significant reservations, as outlined above.

# Response

**Published:** 2018-05-20

**Authors:** Armin Deffur

**Affiliations:** University of Cape Town, South Africa

Many thanks for a thorough review. We made the following changes to address the stated concerns.

 In response to points 1 and 2:

 We added adjusted P-values used as cutoffs in the analytic pipeline in Table 1, and have added a new Table 3, providing detail on the bipartite graphs, based on the recommendation.

 3. Figure 1 has been edited to clarify that both pathways and Chaussabel modules are gene sets forming prior information.

 4. The term "validation study" has been replaced with "example study"

 5. We emphasise in the Results section that the "virtual cells" and the cell matrices are new information we were able to generate based on the shared relationships found in ANIMA

 6. We have added a short section on target audience, in order to clarify use cases for both computational biologists and immunologists

 7. Additional material on comparable tools is provided
